# APP SUMOylation prevents BACE1 cleavage of APP and increases BACE1 degradation to promote the nonamyloidogenic pathway

**DOI:** 10.1186/s10020-025-01354-8

**Published:** 2025-09-29

**Authors:** Yen-Chen Liu, Wei-Lun Hsu, Yun-Li Ma, Chung-Yao Yin, Kuang-Min Cheng, Eminy H. Y. Lee

**Affiliations:** Institute of Biomedical Sciences, Academia Sinica, Taipei, Taiwan

**Keywords:** Amyloid precursor protein, SUMOylation, BACE1, Amyloid-beta, SAPPα, SAPPβ, Alzheimer’s disease

## Abstract

Amyloid precursor protein (APP) plays a key role in the pathogenesis of Alzheimer’s disease (AD). APP undergoes different posttranslational modifications, but the role of SUMOylation modification of APP in the pathogenesis of AD is not known. The molecular mechanism and functional significance of APP SUMOylation have not been studied either. Using in vitro SUMOylation assay, plasmid DNA transfection and lentiviral vector transduction to the mouse hippocampus, we have found that APP is SUMO-modified by Ubc9 at Lys-587 and Lys-595 in the hippocampus endogenously. APP SUMOylation decreases the association between APP and β-secretase (BACE1), reduces amyloid-beta (Aβ), sAPPβ and BACE1 expression, but increases sAPPα expression in APP/PS1 mice. APP SUMOylation also facilitates the degradation of BACE1. Lenti-EGFP-SUMO1 vector transduction to APP/PS1 mice rescues spatial memory and recognition memory deficits, decreases the amount of Aβ and the accumulation of amyloid plaque compared with APP/PS1 mice receiving Lenti-EGFP vector transduction, whereas Lenti-EGFP-SUMO1ΔGG mutant vector transduction to APP/PS1 mice produces an opposite effect for these measures. Melatonin increases Ubc9 expression and enhances APP SUMOylation. In addition, blockade of APP phosphorylation at Thr-668 facilitates APP SUMOylation. These results together suggest that APP SUMOylation promotes the nonamyloidogenic processing of APP and functions as an endogenous protection mechanism against Aβ toxicity. Further, melatonin is an endogenous stimulus that enhances APP SUMOylation.

**Supplementary Information:**

The online version contains supplementary material available at 10.1186/s10020-025-01354-8.

## Introduction

The amyloid precursor protein (APP) is a transmembrane protein that is highly expressed in neurons and is implicated in various synaptic functions (Rodrigues et al. 2014; Wang et al. 2014; Dawson et al. 1999). APP also plays a key role in the pathogenesis of Alzheimer's disease (AD). When APP undergoes the nonamyloidogenic pathway which mainly takes place at the cell membrane, APP is subjected to sequential and proteolytic cleavage by α-secretase and γ-secretase, and sAPPα, COOH-terminal fragment (CTF) C83 and APP intracellular domain (AICD) fragment are generated. The nonamyloidogenic pathway is suggested to play a neuroprotective role against AD in various biological models (Dar and Glazner 2020). Earlier studies have also shown that sAPPα could rescue the deficits in spine density, long-term potentiation and spatial learning in APP knockout mice (Weyer et al. 2014; Ring et al. 2007; Hick et al. 2015). But when APP undergoes the amyloidogenic pathway which mainly occurs in the endosome, APP is subjected to sequential and proteolytic cleavage by β-secretase (BACE1) and γ-secretase; sAPPβ, amyloid-beta (Aβ), C99 and the AICD fragment are generated (De Strooper and Annaert 2000; Chow et al. 2010). Senile plaque is one of the two pathological hallmarks of AD and Aβ peptides are the major components of senile plaques. Amyloid-beta increases lipid peroxidation and free radical production; causes caspase activation and DNA damage that eventually results in neuronal death (Butterfield et al. 2001; Dickson 2004; Hardy and Selkoe 2002). In parallel with these biochemical changes, Aβ also causes cognitive impairment in animals and this cognitive impairment correlates with amyloid plaque formation (Chen et al. 2000; Hsiao et al. 1996) or precedes it (Hsia et al. 1999; Kamenetz et al. 2003). Because BACE1 is essential for generation of the Aβ peptides, BACE1 plays a critical role in Aβ toxicity and AD pathology (Cole and Vassar 2007; Hampel et al., 2021; Sathya et al. 2012).

APP is subjected to a few posttranslational modifications. Several potential phosphorylation sites were identified in the APP cytoplasmic domain. Among these residues, Thr-668 phosphorylaiton was found to play a role in APP metabolism. APP Thr-668 phosphorylation level is increased in hippocampal neurons of the AD brain. Conversely, Aβ production is significantly decreased when APP Thr-668 phosphorylation is abolished (Lee et al. 2003a, b). APP was also found to be ubiquitinated and APP ubiquitination regulates the maturation and degradation of APP (El Ayali et al., (Ayadi, et al., 2012)). In addition to phosphorylation and ubiquitination, neddylation also takes place on APP and conjugation of Nedd8 to the APP C-terminal fragment reduces the interaction of AICD with Fe65 and decreases AICD-mediated transcriptional activation (Lee et al. 2008). More related to the present study, APP was found to be SUMO-modified at Lys-587 and Lys-595 in HeLa cells, and APP SUMOylation decreases Aβ aggregates in cells transfected with AD-associated mutant APP (Zhang and Sarge 2008). In another study, it was found that overexpression of SUMO3 significantly decreases Aβ production whereas overexpression of the dominant-negative SUMO3 mutant increases Aβ production (Li et al. 2003). On the other hand, the SUMO1 protein was found to interact with BACE1 and regulate the level of BACE1 and Aβ. Transfection of *SUMO1* shRNA in BACE1-overexpressed cells was shown to decrease BACE1 expression and Aβ level (Yun et al. 2013). Further, the tau protein was shown as a target of SUMO1 and tau SUMOylation decreases tau degradation and increases tau phosphorylation (Luo et al. 2014). Although these results reveal different roles of AD-associated protein SUMOylation, they all suggest that SUMOylation of a few AD-associated proteins plays a role in the pathogenesis of AD (Dorval and Fraser 2007).

In further examination of the role of protein SUMOylation involved in AD, we have previously found that enhanced SUMOylation of HDAC1, Elk-1 and AICD all plays a protective role against Aβ toxicity in APP/PS1 mice, but their protection mechanisms are different. HDAC1 SUMOylation by the SUMO E3 ligase protein inhibitor of activated STAT1 (PIAS1) reduces HDAC1 association with CREB, increases CREB binding to *Mcl-1* promoter and mediates Aβ induction of Mcl-1 expression to reduce apoptotic neuronal death (Tao et al. 2017). Elk-1 SUMOylation by PIAS1 decreases Elk-1 phosphorylation and down-regulates the expression of the apoptotic gene *GADD45α* to promote neuronal survival (Liu et al. 2019). Moreover, the intracellular domain of APP (AICD) could also be SUMO-modified by PIAS1, and PIAS1 SUMOylation of AICD increases AICD association with CREB and p65 and their DNA binding for transcriptional activation of neprilysin and transthyretin, two major Aβ-degrading enzymes, to reduce Aβ level (Liu et al. 2021). These results suggest that PIAS1 SUMOylation of a few transcription regulators and transcription factor plays a protective role against Aβ toxicity. As mentioned above, APP plays a key role in the pathogenesis of AD, although a previous study has shown that APP could be SUMO-modified in the cell (Zhang and Sarge 2008), whether APP is SUMOylated in the brain, the physiological significance and mechanism of APP SUMOylation in the pathogenesis of AD are not known. In addressing these issues, we have presently found that APP SUMOylation by the SUMO E2 ligase Ubc9 decreases the association between APP and BACE1, reduces Aβ, sAPPβ and BACE1 expression, but increases sAPPα expression in the mouse hippocampus. Thus, APP SUMOylation in the brain has promoted nonamyloidogenic processing of APP and protects against AD.

## Materials and methods

### Animals

Adult male C57BL/6 mice were purchased from the National Laboratory Animal Center, Taiwan. The APP/PS1 mice were purchased from Jackson Laboratory (Bar Harbor, ME) (strain name: B6.Cg-Tg (APPswe, PSEN1dE9)85Dbo/Mmjax, stock number: 005864). Animals of 9–10 month-old were used. For the behavioral experiments, animals received various Lenti-vector transductions at the age around 9 month-old, but when all the behavioral experiments were completed, they were about 10 month-old. All animals were bred and maintained on a 12/12 h light/dark cycle (light on at 8:00 am) at the Animal Facility of the Institute of Biomedical Sciences (IBMS), Academia Sinica with food and water continuously available. Experimental procedures follow the Guidelines of Animal Use and Care of the National Institute of Health and were approved by the Animal Committee of IBMS, Academia Sinica.

### Drugs

Melatonin was purchased from Sigma (Catalog No. M5250, St. Louis, MO). It was dissolved in 100% alcohol and further diluted with PBS to a final concentration of 10 mg/ml in 20% alcohol. The PBS solution contains 20% alcohol for injection to animals in the control group. Cycloheximide was purchased from Sigma (Catalog No. C1988) and dissolved in ethanol to a concentration of 50 mg/ml. Melatonin and cycloheximide were prepared immediately before use.

### Plasmid DNA construction

EGFP-tagged *APP* wild-type plasmid was purchased from Addgene (Catalog No. 69924). QuickChange Site-Directed Mutagenesis Kit (Stratagene, La Jolla, CA) was used to generate the EGFP-tagged *APP*K587RK595R, EGFP-tagged *APP*T668A and EGFP-tagged *APP*T668D mutant plasmids. For construction of EGFP-tagged *sAPP*α and *sAPP*βplasmid, the EGFP-tagged *APP* wild-type plasmid was used as the template, the sequence for the forward primer is 5’-ATCGGTCGACATGCTGCCCGGTTTGGCA-3’. The reverse primer for *sAPP*α is 5’-ATCGGTCGACTTTTTGATGATGAACTTCAT-3’, and that for *sAPPβ* is 5’-ATCGGTCGACCATCTTCACTTCAGAGATC-3’. The PCR product was sub-cloned into the pEGFP-C1 expression vector with *SalI* site. For construction of the EGFP-tagged *PSEN1* plasmid, full-length *PSEN1* was cloned by amplifying the mouse cDNA with primers 5’-ATCGGTCGACATGACAGAGATACCTGCAC-3’ (forward) and 5’-ATCGGTCGACGATATAAAACTGATGGAAT-3’ (reverse). The PCR product was sub-cloned into the pEGFP-C1 expression vector with *SalI* site. Myc-tagged *SUMO1* plasmid and Myc-tagged *SUMO1*ΔGG plasmid were constructed as that described previously (Liu et al. 2021) because SUMO1ΔGG, deletion of two glycine residues from the C-terminal of SUMO1, was shown to prevent the conjugation of SUMO1 to target proteins (Ihara et al. 2005). For construction of EGFP-tagged *SUMO1* plasmid and EGFP-tagged *SUMO1*ΔGG plasmid, the previously cloned Myc-tagged *SUMO1* and Myc-tagged *SUMO1*ΔGG plasmids were used as the templates, and the *SUMO1* and *SUMO1*ΔGG sequences were amplified with the same forward primer 5’-ATCGCGGTCCGATGTCTGACCAGGAGGCA-3’. The reverse primer used for *SUMO1* is: 5’-ATCGCGGACCGCTAAACCGTCGAGTGACC-3’ and that for *SUMO1*ΔGG is: 5’-ATCGCGGACCGCTAAACCGTCGAGTG-3’. The PCR product was sub-cloned into the pEGFP-C1 expression vector with *RsrII* site. For construction of the HA-tagged *Ubc9* plasmid, full-length *Ubc9* was cloned by amplifying the rat *Ubc9* cDNA with primers 5’-GGCGGATCCATGTCGGGGATCGCCCTCAGCAGAC-3’ (forward) and 5’-CATGGAATTCTTATGAGGGCGCAAACTTCTTGG-3’ (reverse). The PCR product was sub-cloned between the *BamHI* and *EcoRI* sites of the expression vector pcDNA3-HA (Invitrogen, Carlsbad, CA).

### Small interference RNA (siRNA)

The sequence for Ubc9 siRNA sense strand is: 5’-AAGCAGAGGCCTACACAATtt-3’ and that for Ubc9 siRNA antisense strand is: 5’-AUUGUGUAGGCCUCUGCUUtt-3’. The Silencer Negative Control number 1 siRNA was used as the control. They were all synthesized from Ambion, Thermo Fisher Scientific (Waltham, MA).

### Lentiviral vector construction and preparation

For construction of EGFP-SUMO1 and EGFP-SUMO1ΔGG lentivitral vectors, full-length EGFP-SUMO1 and EGFP-SUMO1ΔGG cDNA sequences were sub-cloned into the lentiviral vector pLenti-Tri-cistronic (ABM, Richmond, BC, Canada) by amplifying the EGFP-SUMO1 and EGFP-SUMO1ΔGG non-viral expression vectors with different primes. The forward primer used is: 5’-ATCGGGATCCGCCACCATGGTGAGCAAGGGCGAG-3’. The reverse primer used for SUMO1 is: 5’-ATCGCCTAGGCTAAACCGTCGAGTGACC-3’ and that for SUMO1ΔGG is: 5’-ATCGCCTAGGCTAAACCGTCGAGTGCGT-3’. These PCR products were sub-cloned between the *BamHI* and *AvrII* sites of the lentiviral vector.

For lentivirus packaging, HEK293LTV cells (Cell Biolabs, San Diego, CA) were transfected with 9 μg of psPAX2 (Addgene plasmid #12,260), 3 μg of pMD2.G (Addgene plasmid #12,259), and 12 μg of pLenti-EGFP or pLenti-EGFP-SUMO1 or pLenti-EGFP-SUMO1ΔGG plasmid using 40 μl of Lipofectamine 2000 (Invitrogen) in 10-cm cell culture dish. Lentiviral particles were collected using the speedy lentivirus purification solution (ABM) according to the manufacturer's protocols, and the viral stock was stored at −80 °C in aliquots. The lentivirus titer was determined by using the lentivirus qPCR Titer Kit (ABM) according to the manufacturer's protocols (ABM). The lentiviral particles were re-suspended in ice-cold PBS and the final concentration of the lentiviral vector used for hippocampal injection is 5 × 10^8^ IU/ml.

### Cell culture and plasmid transfection

HEK293T cell were maintained in Dulbecco’s modified Eagle’s medium containing 10% fetal bovine serum and incubated at 37 °C in a humidified atmosphere with 5% CO_2_. Plasmid transfection was made by using the Lipofectamine 2000 reagent in 6-well culture plates according to the manufacturer’s instructions. Immunoprecipitation (IP) and western blot were conducted 48 h after plasmid transfection.

### Plasmid DNA and siRNA transfection to the hippocampus

Mice were anesthetized with pentobarbital (40 mg/kg, i.p.) and subjected to stereotaxic surgery. A total of 0.25 μl was injected to each side of the CA1 area at a rate of 0.1 μl/min. Transient plasmid DNA and siRNA transfections were conducted using the non-viral transfection agent polyethyleneimine (PEI) (0.1 mM), and we have previously demonstrated that PEI does not produce toxicity to hippocampal neurons (Chao et al. 2011). Briefly, plasmid DNA was diluted in 5% glucose to a stock concentration of 2.77 μg/μl. Branched PEI of 25 kDa (Sigma) was diluted to 0.1 mM concentration in 5% glucose and added to the DNA solution. Immediately before injection, 0.1 mM PEI was added to reach a ratio of PEI nitrogen per DNA phosphate equals to 10. The mixture was subjected to vortex for 30 s and allowed to equilibrate for 15 min. For siRNA injection, 0.25 μl of Ubc9 siRNA (10 pmol) and control siRNA was transfected to the mouse CA1 area bilaterally also using the transfection agent PEI. The sequence for Ubc9 siRNA sense strand is: 5’-AAGCAGAGGCCTACACAATtt-3’ and that for Ubc9 siRNA antisense strand is: 5’-AUUGUGUAGGCCUCUGCUUtt-3’. The Negative Control siRNA was used as the control. They were all synthesized from Ambion, Thermo Fisher Scientific. The injection needle was left in place for 5 min to limit the diffusion of injected agent. Animals were sacrificed 48 h after plasmid transfection or siRNA injection and their hippocampal tissue was dissected out and subjected to co-immunoprecipitation, western blot and in vitro SUMOylation assay.

### In vitro SUMOylation assay

Hippocampal CA1 tissue lysate was prepared for SUMOylation assay in the same way as that prepared for western blot. For immunoprecipitation (IP) experiment, the clarified lysate (0.5 mg) was immunoprecipitated with 3 μl of anti-EGFP antibody at 4 °C overnight. The protein A agarose beads (30 ml, 50% slurry, GE Healthcare, IL) were added to the IP reaction product to catch the immune complex at 4 °C for 3 h. The immune complex on beads were washed three times with washing buffer containing 20 mM HEPES (pH 7.4), 150 mM NaCl, 1 mM EDTA, 1% IGEPAL CA-630, 1 mM DTT, 50 mM β-glycerophosphate, 50 mM NaF, 10 mg/ml PMSF, 4 mg/ml aprotinin, 4 mg/ml leupeptin and 4 mg/ml pepstatin and subjected to in vitro SUMOylation reaction with the addition of the E1 (1 μL), E2 (1 μL), and the SUMO1 (0.5 μL) proteins provided in the SUMOlink kit (Active Motif, CA, USA). In vitro SUMOylation assay was performed using the SUMO linkTM kit according to the manufacturer’s instructions (Active Motif, CA) and boiled in Laemmli sample buffer at 95 °C for 10 min. The SUMOylation reaction product was subjected to 8% SDS-PAGE and transferred onto the PVDF membrane. The membrane was immunoblotted with anti-SUMO1 antibody (1:3000; Active Motif) or anti-EGFP antibody (1:8000; Sigma-Aldrich; Catalog No. 11814460001). For determination of endogenous APP SUMOylation after Ubc9 siRNA transfection, the clarified lysate (0.5 mg) was immunoprecipitated with 3 μL of anti-APP C-terminal fragment antibody (BioLegend) at 4 °C overnight. The protein A magnetic beads (30 mL, 50% slurry, GE Healthcare, IL, USA) were added to the IP reaction product to catch the immune complex at 4 °C for 3 h. The immune complex on beads was washed three times with washing buffer and recombinant E1 and SUMO1 (without E2) proteins were added to the IP reaction product. The remaining procedures were the same as that described above.

### Immunoprecipitation (IP) and western blot

Cell lysate and mouse tissue were lysed by brief sonication in RIPA lysis buffer containing 50 mM Tris–HCl (pH 7.4), 150 mM NaCl, 2 mM EDTA, 1% IGEPAL CA-630 and 20 mM N-ethylmaleimide (Catalog No. E3876-5G, Sigma-Aldrich, Darmstadt, Germany). One tablet of protease inhibitor cocktail (Catalog No. 05892791001, cOmplete ULTRA Tablets, Mini, EDTA-free, EASYpack, Roche, Mannheim, Germany) and one tablet of phosphatase inhibitor (Catalog No. 04906837001, PhosSTOP, Roche) were added to each 10 ml of the RIPA lysis buffer. For IP of full-length APP, BACE1 and EGFP, the clarified lysate (0.5 mg) was immunoprecipitated with 3 μl of anti-APP C-terminal fragment antibody (Catalog No. 802801, BioLegend, San Diego, CA), 3 μl of anti-BACE1 antibody (Catalog No. 5606, Cell Signaling, Danvers, MA) or 3 μl of anti-GFP antibody (Catalog No. 11814460001, Sigma-Aldrich) at 4 °C for overnight. The protein A or G magnetic beads (30 μl, 50% slurry, GE Healthcare, Chicago, IL) were added to the IP reaction product to catch the immune complex at 4 °C for 3 h. The immune complex on beads were washed three times with washing buffer containing 20 mM HEPES (pH 7.4), 150 mM NaCl, 1 mM EDTA, 1% IGEPAL CA-630, 1 mM DTT, 50 mM β-glycerophosphate, 50 mM NaF, 10 mg/ml PMSF, 4 μg/ml aprotinin, 4 μg/ml leupeptin and 4 μg/ml pepstatin and were subjected to 6% or 8% SDS-PAGE followed by transferring onto the PVDF membrane (Catalog No. IPVH00010, Millipore, Bedford, MA). Western blot was conducted using the following antibodies: anti-APP C-terminal fragment antibody (1:3000, BioLegend), anti-SUMO1 antibody (1:3000, Catalog No. 4930, Cell Signaling), anti-BACE1 antibody (1:3000, Cell Signaling), anti-GFP antibody (1:8000; Sigma-Aldrich), anti-Ubc9 antibody (1:3000, Catalog No. ab75854, Abcam, Cambridge, UK), anti-sAPPα antibody (1:1000, Catalog No. ab15272, Abcam), anti-sAPPα antibody (1:2000, Catalog No. 11088, IBL, Minneapolis, MN), anti-sAPPβantibody (1:1000, Catalog No. SIG-39138, BioLegend), anti-APP antibody (22C11) (1:3000, Catalog No. 14–9749-82, Thermo Fisher), anti-Myc antibody (1:6000, Catalog No. 05–419, Millipore), anti-HA antibody (1:6000, Catalog No. 05–904, Millipore), anti-phospho-APPT668 antibody (1:5000, Catalog No. AP0200, Abclonal, Woburn, MA), anti-β-amyloid antibody (1:1000, Catalog No. 803001, BioLegend), anti-phospho-MAPK (pERK1/2) antibody (1:5000, Catalog No. 4376, Cell Signaling), anti-MAPK (ERK1/2) antibody (1:5000, Catalog No. 4695, Cell Signaling) and anti-actin (1:200,000, Catalog No. MAB1501, Millipore) antibody. The secondary antibody used was HRP-conjugated goat-anti rabbit IgG antibody or HRP-conjugated goat-anti mouse IgG antibody (1:8000, Catalog No. 111–035-003 and 115–035-003, Jackson ImmunoResearch, West Grove, PA). The secondary antibody used for co-IP experiment was HRP-conjugated goat-anti rabbit IgG light chain antibody (1:6000, Catalog No. 112–035-175, Jackson ImmunoResearch) or HRP-conjugated goat-anti mouse IgG light chain antibody (1:6000, Catalog No. 115–035-174, Jackson ImmunoResearch). Membrane was developed by reacting with chemiluminescence HRP substrate (Millipore) and was exposed to the LAS-3000 image system (Fujifilm, Tokyo, Japan) for visualization of protein bands. The protein bands were quantified by using the NIH Image J Software.

#### Immunohistochemistry

For immunohistochemical staining of Ubc9 and full-length APP, TrkB and full-length APP as well as Rab5 with full-length APP, in the CA1 area of the mouse brain, mice were anesthetized with pentobarbital (100 mg/kg, i.p.) and perfused with ice-cold phosphate-buffered saline followed by 4% paraformaldehyde. Brains were removed and post-fixed in 30% sucrose/4% paraformaldehyde solution for 48 h. Brains were then frozen, cut into 30-μm sections on a cryostat and mounted on gelatin-coated slides. Brain sections were rinsed with PBS for 10 min and antigen was retrieved with 0.1 M citric acid/0.1 M sodium citrate buffer at 95 °C for 45 min followed by PBS wash for 10 min for three times. The sections were pre-incubated in a blocking solution containing 3% BSA and 0.5% Triton X-100 in PBS for 1 h. For visualization of endogenous Ubc9 and full-length APP in hippocampal CA1 neurons, brain sections were incubated with rabbit anti-Ubc9 antibody (1:200, Catalog No. ab75854, Abcam) and mouse anti-APP C-terminal fragment antibody (1:200, Catalog No. 802801, BioLegend) at 4 °C for overnight. Brain sections were then washed with PBS for 10 min for three times and incubated with goat anti-mouse antibody conjugated with Alexa Fluor 488 (1:500, Catalog No. 115–545-146, Jackson ImmunoResearch Laboratories) and goat anti-rabbit antibody conjugated with Alexa Fluor 594 (1:500; Jackson, Catalog No. 111–585-144, ImmunoResearch Laboratories) for 1 h and then washed with PBS for 10 min for three times. For visualization of endogenous TrkB and full-length APP in hippocampal CA1 neurons, brain sections were incubated with mouse anti-TrkB antibody (1:50, Catalog No. sc-377218, Santa Cruz) and rabbit anti-amyloid precursor protein antibody (1:200, Catalog No. ab32136, Abcam) at 4 °C for overnight. Brain sections were then washed with PBS for 10 min for three times and incubated with goat anti-rabbit antibody conjugated with Alexa Fluor 488 (1:500, Catalog No. 111–545-144, Jackson ImmunoResearch Laboratories) and goat anti-mouse antibody conjugated with Alexa Fluor 594 (1:500; Jackson, Catalog No. 115–585-146, ImmunoResearch Laboratories) for 1 h and then washed with PBS for 10 min for three times. For visualization of endogenous Rab5 and full-length APP in hippocampal CA1 neurons, brain sections were incubated with rabbit anti-Rab5 antibody (1:200, Catalog No. ab109534, Abcam) and mouse anti-APP C-terminal fragment antibody (1:200, Catalog No. 802801, BioLegend) at 4 °C for overnight. Brain sections were then washed with PBS for 10 min for three times and the secondary antibodies used were the same as that described for staining with the anti-Ubc9 and anti-APP C-terminal fragment antibodies. For immunofluorescence detection of the nucleus, tissue sections were added with 20 μl of the DAPI Fluoromount-G mounting medium (SouthernBiotech, Birmingham, AL). For immunofluorescence detection of amyloid plaque in the CA1 area, mice were anesthetized with pentobarbital (100 mg/kg, i.p.) followed by the same procedures as described above. For visualization of endogenous amyloid plaque in APP/PS1 mice, brain sections containing the CA1 area were incubated with ProteoStat Amyloid Plaque Detection Kit (Enzo Life Sciences; Catalog No. ENZ-51035) for 30 min and washed with PBS for 10 min for three times at room temperature. This detection kit was used because we have demonstrated that ProteoStat dye staining well co-localizes with anti-amyloid-beta staining in the hippocampus (Supplementary Fig. 1). The brain sections were then mounted with 20 μl DAPI Fluoromount-G mounting medium (SouthernBiotech). Photomicrographs were taken using a Zeiss LSM700 confocal microscope (Carl Zeiss, Oberkochen, Germany). The number of plaques showing ProteoStat dye staining was counted by using the NIH Image J Software.

### Water maze learning

The water maze used was a plastic, circular pool, 1.2 m in diameter and 25 cm in height that was filled with water (25 ± 2 °C) to a depth of 16 cm. A circular platform of 8 cm in diameter was placed at a specific location away from the edge of the pool. The top of the platform was submerged 0.6 cm below the water surface. Water was made cloudy by adding milk powder. Distinctive, visual cues were set on the wall. For spatial learning, animals were subjected to three trials a day with one given early in the morning, one given in the early afternoon and the other one given in the late afternoon. The training procedure lased for 5 days and a total of 15 trials were given. For these trials, animals were placed at different starting positions spaced equally around the perimeter of the pool in a random order. Animals were given 60 s to find the platform. If an animal could not find the platform within 60 s, it was guided to the platform and was allowed to stay on the platform for 20 s. The time that each animal took to reach the platform was recorded as the escape latency. A probe trial of 60 s was given on day 6 to test their memory retention. Animals were placed in the pool with the platform removed and the time they spent in each quadrant (target quadrant, left quadrant, opposite quadrant and right quadrant) as well as the total distance travelled in the target quadrant were recorded.

### Novel object recognition learning

The procedures used for novel object recognition learning was adopted from that of a previous study with slight modifications (Lee et al. 2003a, b). Animals were habituated to an open field box (35 × 35 × 35 cm) individually for 10 min one day before the experiment. During training, two different novel objects were placed in the open field box and the animal was allowed to explore the objects for 10 min. The criteria used for exploration were a distance less than 1.5 cm between the animal and the object or a direct contact of the animal to the object. During the retention test given 5 h later, the animal was placed back to the same box but one of the familiar objects was replaced with a novel object of approximately the same size. The time each animal spent exploring the two objects during the 10 min period was also recorded. Exploratory preference was calculated as the ratio of time animals spent exploring each object to that of exploring both objects during the training and retention tests, respectively.

### Statistical analysis

Spatial acquisition (escape latency) data were analyzed with two-way analysis of variance (ANOVA) with repeated measure followed by post-hoc Newman-Keuls multiple comparisons (represented by q value). Retention performance data, novel object recognition memory data and biochemical data were analyzed with the Student’s t-test or one-way ANOVA followed by Newman-Keuls comparisons. Values of *p* < 0.05 were considered statistically significant (^*^ *P* < 0.05, ^**^ *P* ≤ 0.01, ^#^ *P* < 0.001).

## Results

### APP is SUMO-modified by Ubc9 at Lys-587 and Lys-595 in the hippocampus

**Fig. 1:**
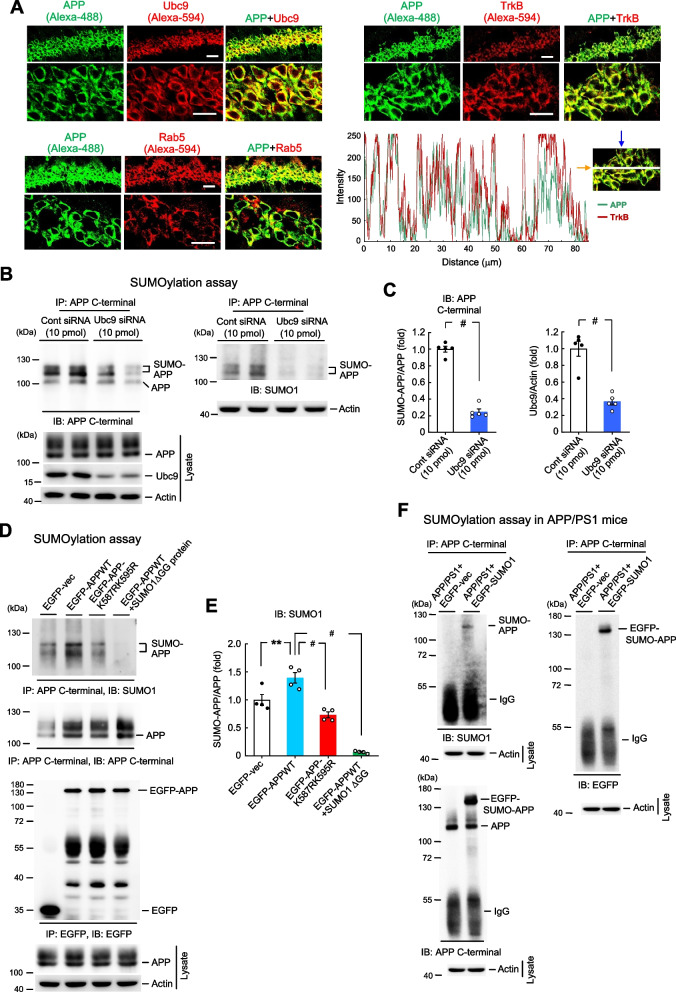
APP is SUMO-modified by Ubc9 at Lys-587 and Lys-595 in the hippocampus. **A** Immunohistochemistry showing the distribution of APP and Ubc9 and their co-localization on the cell membrane of hippocampal neurons (*n* = 3), upper-left panel. Distribution of APP and TrkB and their co-localization on the cell membrane of hippocampal neurons (*n* = 3), upper-right panel. Distribution of APP and Rab5 and their co-localization in the same hippocampal neurons (*n* = 3), lower-left panel. Scale bar is 40 μm for the upper panels and scale bar is 20 μm for the lower panels. Line scan analysis shows the co-localization of APP and the membrane marker TrkB on the cell membrane of hippocampal neurons, lower-right panel. **B** Ubc9 siRNA (10 pmol) or control siRNA was transfected to the mouse CA1 area and endogenous APP SUMOylation was determined 48 h later by SUMOylation assay. APP and Ubc9 expression in the lysate was determined by western blot (*n* = 5). IP: immunoprecipitation, IB: immunoblotting. **C** The quantified results of APP SUMOylation (from upper-left panel of B) and Ubc9 expression (from lower-left panel of B) are shown (t_1,8_ = 15.53 for APP SUMOylation and t_1,8_ = 6.46 for Ubc9 expression, both *P* < 0.001). **D** EGFP-vector, EGFP-APPWT (with or without the SUMO1ΔGG mutant protein added to the reaction) or EGFP-APPK587RK595R mutant plasmid was transfected to mouse CA1 area and SUMOylation assay was carried out 48 h later. Co-IP with anti-EGFP antibody was conducted to confirm the expression of the plasmids. The amount of input APP is also shown (*n* = 4). **E** The quantified result of APP SUMOylation is shown (F_3,12_ = 56.59, *P* < 0.001; q = 5.32, *P* < 0.01 comparing the EGFP-APPWT group with EGFP-vector group; q = 8.9, *P* < 0.001 comparing the EGFP-APPK587RK595R group with EGFP-APPWT group; q = 17.89, *P* < 0.001 comparing the EGFP-APPWT + SUMO1ΔGG protein group with EGFP-APPWT group). **F** EGFP-vector or EGFP-SUMO1 plasmid was transfected to the hippocampus of APP/PS1 mice and APP SUMOylation assay was carried out 48 h later (IP with anti-APP C-terminal antibody and IB with anti-SUMO1 antibody for upper-left panel; IP and IB with anti-APP C-terminal antibody for lower-left panel; IP with anti-APP C-terminal antibody and IB with anti-EGFP antibody for upper-right panel). Experiments are in two repeats. Data are mean ± SEM. ** *P* < 0.01, ^**#**^ *P* < 0.001

In this series of experiments, we examined whether APP can be SUMO-modified by Ubc9 in the hippocampus and the candidate SUMOylation residues on APP. We first examined whether APP co-localizes with Ubc9 in the same neurons. Mouse hippocampal tissue slices were subjected to immunohistochemistry staining for APP and Ubc9. Results revealed that APP is mainly expressed on the cell membrane and Ubc9 is mainly expressed in the cytosol area including the cell membrane. Further, APP and Ubc9 are co-localized in the same neurons on the cell membrane only (Fig. 1A, upper-left panel). To confirm that APP and Ubc9 do co-localize on the cell membrane, we have adopted another membrane marker TrkB, the receptor for brain-derived neurotrophic factor in the brain, and examined the co-localization of APP and TrkB. Our result revealed that APP and TrkB are well co-expressed in the same hippocampal neurons (Fig. 1A, upper-right panel) and this co-localization is verified by line scan analysis (Fig. 1A, lower-right panel). Because APP also localizes to endosome and undergoes amyloidogenic processing, we have adopted the endosome marker Rab5 and examined the cellular localization of APP and Rab5. Immunohistochemical result revealed that APP is co-localized with Rab5 in some of the hippocampal neurons (Fig. 1A, lower-left panel).

Next, we examined whether APP is SUMO-modified by Ubc9 endogenously in the brain. Control siRNA or Ubc9 siRNA was transfected to the mouse hippocampus and animals were sacrificed 48 h later. Their hippocampal tissue was subjected to SUMOylation assay for determination of endogenous APP SUMOylation. Results indicated that Ubc9 siRNA transfection significantly decreased the level of endogenous APP SUMOylation in the hippocampus when the immunoprecipitation product is immunoblotted with either the anti-APP C-terminal antibody or the anti-SUMO1 antibody (Fig. 1B). Ubc9 siRNA transfection also markedly decreased Ubc9 expression level, confirming the effectiveness of Ubc9 siRNA transfection (Fig. 1B, lower panel). The quantified results of APP SUMOylation and Ubc9 expression are shown in Fig. 1C. APP SUMOylation level was determined based on the result from Fig. 1B, the upper-left panel.

Next, we determined the candidate SUMOylation residues on APP. A previous study has shown that APP is SUMO-modified at Lys-587 and Lys-595 in HeLa cells (Zhang and Sarge 2008), but it is not known whether APP is SUMO-modified at the same residues in the brain. This issue was examined here. We first generated the Lys-587 and Lys-595 sumo-mutants, K587R and K595R, respectively. Wild-type (WT) mice were divided to four groups and received the following transfections, respectively, EGFP-vector, EGFP-APPWT plasmid, EGFP-APPK587RK595R double sumo-mutant plasmid and EGFP-APPWT plasmid but with the SUMO1ΔGG mutant protein added to the SUMOylation reaction. Animals were sacrificed 48 h after plasmid transfection and their hippocampal tissue was subjected to the SUMOylation assay. Results revealed that APP is consistently SUMO-modified when the EGFP-APPWT plasmid was transfected, but APP SUMOylation was apparently reduced when the EGFP-APPK587RK595R double sumo-mutant plasmid was transfected. APP SUMOylation was completely abolished when the SUMO1ΔGG mutant protein was added to the reaction (Fig. 1D, upper panel). The quantified result of APP SUMOylation is shown in Fig. 1E. The same cell lysates were also immunoprecipitated with anti-EGFP antibody and immunoblotted with anti-EGFP antibody to confirm plasmid transfection and expression in the hippocampus (Fig. 1D, lower panel).

The above results indicated that APP is SUMO-modified at Lys-587 and Lys-595 when the APPWT plasmid is transfected to the hippocampus, next we examined whether APP is SUMO-modified in the brain of APP/PS1 mice endogenously. APP/PS1 were divided to two groups and received EGFP-vector or EGFP-SUMO1 plasmid transfection to their hippocampus. They were sacrificed 48 h later and their hippocampal tissue was subjected to SUMOylation assay for determination of endogenous APP SUMOylation. Cell lysates were first immunoprecipitated with anti-APP C-terminal antibody and immunoblotted with anti-SUMO1 antibody. Results revealed that the APP SUMOylation band is apparently observed in APP/PS1 mice transfected with EGFP-SUMO1 plasmid, but not in APP/PS1 mice transfected with EGFP-vector plasmid (Fig. 1F, upper-left panel). The same cell lysates were also immunoblotted with anti-APP C-terminal antibody and the result similarly indicated that the EGFP-APP SUMOylation band was only observed in APP/PS1 mice transfected with EGFP-SUMO1 plasmid, but not in APP/PS1 mice transfected with EGFP-vector plasmid (Fig. 1F, lower-left panel). The same cell lysates were then immunoblotted with anti-EGFP antibody to confirm that the EGFP-APP SUMOylation band was only observed in APP/PS1 mice transfected with the EGFP-SUMO1 plasmid (Fig. 1F, right panel).

### APP SUMOylation decreases the association between APP and BACE1, reduces Aβ, sAPPβ and BACE1 expression, but increases sAPPα expression in APP/PS1 mice

**Fig. 2:**
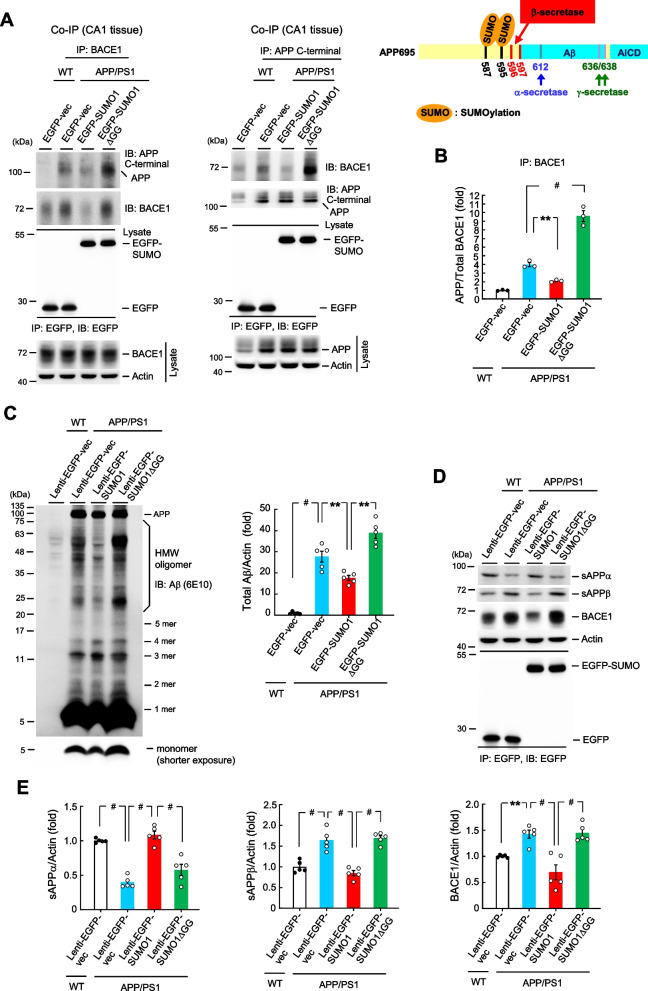
APP SUMOylation decreases the association between APP and BACE1, reduces Aβ, sAPPβ and BACE1 expression, but increases sAPPα expression in APP/PS1 mice. **A** EGFP-vector or EGFP-SUMO1 or EGFP-SUMO1ΔGG plasmid was transfected to the hippocampus of WT or APP/PS1 mice. Forty-eight hours later, their CA1 tissue lysate was immunoprecipitated with anti-BACE1 antibody and immunoblotted with anti-APP C-terminal antibody and anti-BACE1 antibody (left) and *vise versa* (right). The same cell lysate was also immunoprecipitated and immunoblotted with anti-EGFP antibody (lower panels) (*n* = 3). The locations of SUMOylation residues and BACE1 cleavage site on the APP protein are shown (upper-right panel). **B** The quantified result of normalized APP over BACE1 expression obtained from Fig. 2 A, upper-left panel (F_3,8_ = 126.1, *P* < 0.001; q = 5.55, *P* < 0.01 comparing the EGFP-vector group and EGFP-SUMO1 group in APP/PS1 mice; q = 16.49, *P* < 0.001 comparing the EGFP-vector group with EGFP-SUMO1ΔGG group in APP/PS1 mice). **C** Lenti-EGFP-vector or Lenti-EGFP-SUMO1 or Lenti-EGFP-SUMO1ΔGG plasmid was transducted to the hippocampus of WT or APP/PS1 mice. Forty-eight hours later, their CA1 tissue lysate was subjected to western blot determination of Aβ and Aβ oligomerization expression (left panel) (*n* = 5). The quantified result is shown in the right panel (F_3,16_ = 71.18, *P* < 0.001; q = 5.0, *P* < 0.001 comparing Lenti-EGFP-vector in APP/PS1 mice with Lenti-EGFP-vector in WT mice; q = 3.55, *P* < 0.01 comparing Lenti-EGFP-SUMO1 in APP/PS1 mice with Lenti-EGFP-vector in APP/PS1 mice; q = 4.8, *P* < 0.01 comparing Lenti-EGFP-SUMO1ΔGG in APP/PS1 mice with Lenti-EGFP-SUMO1 in APP/PS1 mice). **D** The same tissue lysate was also subjected to western blot determination of sAPPα, sAPPβ and BACE1 expression (*n* = 5). IP and IB with anti-EGFP antibody were conducted to verify various Lenti-EGFP vector transduction and expression (lower panel). **E** The quantified results of sAPPα, sAPPβ and BACE1 expression (F_3,16_ = 38.46, *P* < 0.001; q = 11.32, *P* < 0.001 comparing Lenti-EGFP-vector in APP/PS1 mice with Lenti-EGFP-vector in WT mice; q = 12.89, *P* < 0.001 comparing Lenti-EGFP-SUMO1 in APP/PS1 mice with Lenti-EGFP-vector in APP/PS1 mice; q = 9.51, *P* < 0.001 comparing Lenti-EGFP-SUMO1ΔGG in APP/PS1 mice with Lenti-EGFP-SUMO1 in APP/PS1 mice for sAPPα) (F_3,16_ = 30.36, *P* < 0.001; q = 8.16, *P* < 0.001 comparing Lenti-EGFP-vector in APP/PS1 mice with Lenti-EGFP-vector in WT mice; q = 10.09, *P* < 0.001 comparing Lenti-EGFP-SUMO1 in APP/PS1 mice with Lenti-EGFP-vector in APP/PS1 mice; q = 10.74, *P* < 0.001 comparing Lenti-EGFP-SUMO1ΔGG in APP/PS1 mice with Lenti-EGFP-SUMO1 in APP/PS1 mice for sAPPβ) (F_3,16_ = 16.02, *P* < 0.001; q = 4.69, *P* < 0.01 comparing Lenti-EGFP-vector in APP/PS1 mice with Lenti-EGFP-vector in WT mice; q = 8.07, *P* < 0.001 comparing Lenti-EGFP-SUMO1 in APP/PS1 mice with Lenti-EGFP-vector in APP/PS1 mice; q = 8.32, *P* < 0.001 comparing Lenti-EGFP-SUMO1ΔGG in APP/PS1 mice with Lenti-EGFP-SUMO1 in APP/PS1 mice for BACE1). Data are mean ± SEM. ** *P* < 0.01, ^**#**^ *P* < 0.001

The above results showed that APP could be SUMO-modified in the brain endogenously. Here we examined the molecular mechanism and physiological significance of APP SUMOylation. BACE1 is the key enzyme that cleaves the APP protein to generate Aβ and promotes the amyloidogenic pathway. BACE1 was found to cleave APP between Met-596 and Asp-597 (Roher et al. 1993; Kimura et al. 2016). Because the SUMOylation sites and the BACE1 cleavage site are located very closely on the APP protein (Fig. 2A, right panel), it is conceivable that SUMOylation of APP may affect BACE1 association with APP and subsequent cleavage of APP and vice versa. To test this hypothesis, we first examined whether SUMOylation of APP alters the association between APP and BACE1 by conducting the co-IP experiments. The EGFP vector plasmid was transfected to the hippocampus of WT mice and different EGFP-tagged plasmids were transfected to the hippocampus of APP/PS1 mice. Animals were sacrificed two days later and their hippocampal tissues were immunoprecipitated with anti-BACE1 antibody and immunoblotted with anti-APP C-terminal antibody and anti-BACE1 antibody. Results revealed that the association between BACE1 and APP is almost not observed in WT mice. BACE1 is apparently associated with APP in APP/PS1 mice, but this association is reduced when the EGFP-SUMO1 plasmid was transfected to the APP/PS1 mice. Conversely, this association is increased when the EGFP-SUMO1ΔGG plasmid was transfected to the APP/PS1 mice (Fig. 2A, upper-left panel). Immunoprecipitation and immunoblotting with anti-EGFP antibody confirms the transfection and expression of these EGFP-tagged plasmids (Fig. 2A, lower-left panel). The level of BACE1 in total lysate was also shown (Fig. 2A, lower-left panel). The same results were obtained when these tissue lysates were immunoprecipitated with anti-APP C-terminal antibody and immunoblotted with anti-BACE1 antibody (Fig. 2A, upper-middle panel). EGFP-tagged plasmid transfection and expression was also confirmed by immunoprecipitation and immunoblotting with anti-EGFP antibody (Fig. 2A, lower-middle panel). The level of APP in total lysate was also shown (Fig. 2A, lower-middle panel). In this experiment, the binding of BACE1 to APP is decreased in the SUMO1 overexpression group. To avoid the possibility that it is due to decreased level of BACE1 in this group, APP association with BACE1 is expressed as the normalized ratio of APP over total BACE1 from the result obtained in Fig. 2A, the upper-lower panel, and is shown in Fig. 2B.

Next, we examined the role of APP SUMOylation in the generation of Aβ. A different batch of WT mice and APP/PS1 mice received different Lenti-EGFP vector transductions and they were sacrificed 14 days later. Their hippocampal tissue was subjected to western blot determination of Aβ, sAPPα, sAPPβ and BACE1 expression. Results indicated that Aβ is almost not observed in WT mice receiving Lenti-EGFP vector transduction. A fair amount of Aβ and Aβoligomerization was observed in APP/PS1 mice receiving Lenti-EGFP vector transduction. Amyloid-β and Aβ oligomerization were apparently decreased by Lenti-EGFP-SUMO1 transduction in APP/PS1 mice. However, Aβ and Aβ oligomerization were increased in APP/PS1 mice receiving Lenti-EGFP-SUMO1ΔGG transduction compared with APP/PS1 mice receiving Lenti-EGFP-SUMO1 transduction (Fig. 2C, left panel). The quantified result of total Aβ expression is shown in the right panel of Fig. 2C. In addition, the expression level of sAPPβ and BACE1 parallels with that of Aβ expression, but the expression level of sAPPα is in the opposite direction (Fig. 2D). Immunoprecipitation and immunoblotting with anti-EGFP antibody confirms the transduction and expression of these lentiviral vectors (Fig. 2D, lower panel). The quantified results of sAPPα, sAPPβ and BACE1 expression are shown in Fig. 2E. Because the 6E10 Aβ antibody also recognizes APPCTFβ (C99) and sAPPα, in order to validate the above results, we have also used the APP C-terminal antibody and 22C11 antibody. Result from western blotting revealed that the APP C-terminal antibody recognizes the full-length APP, the APPCTFβ and APP CTFα (C83). The alternation of C99 expression is consistent with that of Aβ expression in Fig. 2C and the C99 band is possibly confounded with the 3-mer Aβ band (~ 11 kDa) observed in Fig. 2C, but not the Aβ monomer and other Aβoligomer bands (Supplementary Fig. 2 A). Therefore, it should not apparently affect the quantified result of total Aβ expression in Fig. 2C (right panel). Although the 6E10 antibody also recognizes sAPPα, however, the sAPPα band should be around 90–95 kDa in size and it was not observed in our Aβ oligomerization assay in Fig. 2C; therefore, it should not affect the quantification of total Aβ expression level either. The 22C11 antibody is supposed to recognize the full-length APP, sAPPα and sAPPβ. Our result of using the 22C11 antibody revealed similar results as that shown in Fig. 2D in terms of sAPPβ expression, but this antibody did not recognize sAPPα (Supplementary Fig. 2B).

To further validate the specific and reliable detection of sAPPα and sAPPβ, we have generated the sAPPα and sAPPβ clones and overexpressed them in HEK293T cells, respectively. We then used the sAPPα and sAPPβ antibodies adopted in Fig. 2D to carry out western blot assay. Results showed that the sAPPα antibody specifically recognized the sAPPα band (Supplementary Fig. 2 C) and the sAPPβ antibody specifically recognized the sAPPβ band (Supplementary Fig. 2D).

### APP SUMOylation facilitates BACE1 degradation

**Fig. 3:**
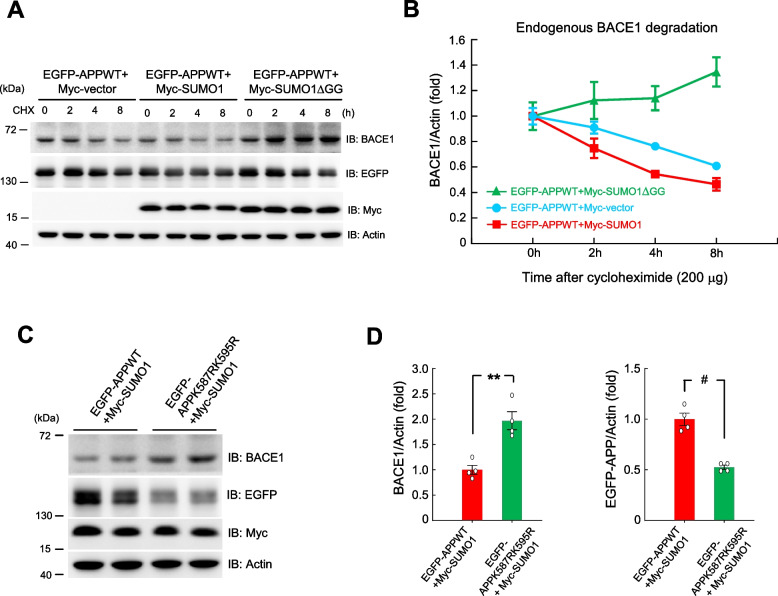
APP SUMOylation facilitates BACE1 degradation. **A** EGFP-APPWT plasmid was co-transfected with Myc-vector, Myc-SUMO1 or Myc-SUMO1ΔGG plasmid to HEK293T cells (200 ng per well). Cycloheximide (200 μg) was added to the cell 24 h after plasmid transfection for different time periods. Cell lysates were prepared with western blot determination of BACE1, EGFP and Myc expression. *n* = 3 for each time point for each group. **B** The quantified results of BACE1 expression over time for each group are shown. Experiments are in three repeats (F_2,6_ = 10.85, *P* = 0.01. **C** EGFP-APPWT or EGFP-APPK587RK595R plasmid was co-transfected with Myc-SUMO1 plasmid to HEK293T cells and western blot determination for BACE1, EGFP and Myc expression was determined 48 h later (*n* = 4 each group). **D** The quantified results of BACE1 expression (left panel) and EGFP-APP expression (right panel) (t_1,6_ = 4.87, *P* < 0.01 for BACE1 and t_1,6_ = 7.45, *P* < 0.001 for EGFP-APP). Data are mean ± SEM. ** *P* ≤ 0.01, ^**#**^ *P* < 0.001

Results from Fig. 2D indicated that transduction of Lenti-EGFP-SUMO1 vector to APP/PS1 mice decreases the expression of BACE1. Here, we examined whether SUMOylation of the APP protein affects the stability of BACE1. EGFP-APPWT plasmid was co-transfected with Myc-vector plasmid, Myc-SUMO1 plasmid or Myc-SUMO1ΔGG plasmid to HEK293T cells with cycloheximide added to the cell for different time periods. The cell lysates were subjected to western blot determination of BACE1 expression. EGFP and Myc expression level was also determined to confirm the transfection of these plasmids (Fig. 3A). BACE1 expression at different time points after cycloheximide treatment for these three groups is shown in Fig. 3B. The results indicated that co-transfection of EGFP-APPWT and Myc-SUMO1 plasmids facilitates the degradation of BACE1 whereas co-transfection of EGFP-APPWT and Myc-SUMO1ΔGG plasmids slows down the degradation of BACE1.

Next, we examined whether the above result is indeed due to SUMOylation of the APP protein. To examine this issue, EGFP-APPWT plasmid or EGFP-APPK587RK595R sumo-mutant plasmid was co-transfected with Myc-SUMO1 plasmid to HEK293T cells and western blot was carried out to determine the expression level of BACE1, EGFP and Myc 48 h later. Results indicated that APP sumo-mutant plasmid transfection markedly increases BACE1 expression (Fig. 3C and D, left panel), but it decreases EGFP-APP expression (Fig. 3C and D, right panel).

### Lenti-EGFP-SUMO1 transduction to APP/PS1 mice rescues spatial learning and memory deficits compared with APP/PS1 mice receiving Lenti-EGFP vector transduction

**Fig. 4:**
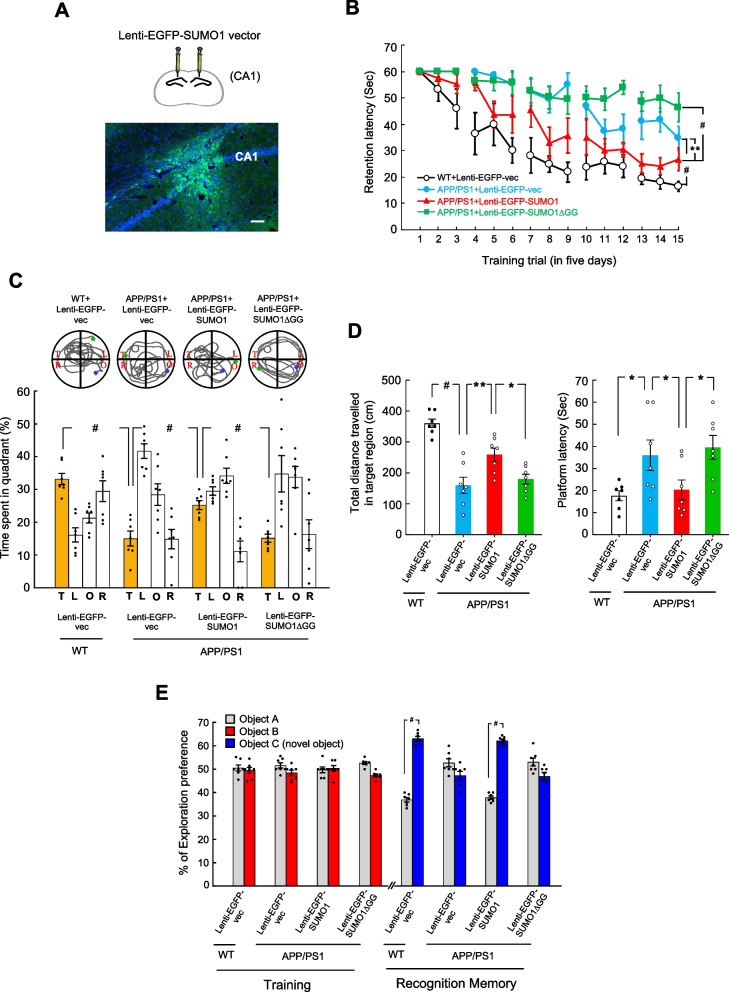
Lenti-EGFP-SUMO1 transduction to APP/PS1 mice rescues spatial memory and recognition memory deficits compared with APP/PS1 mice receiving Lenti-EGFP vector transduction. **A** Illustration and immunohistochemistry showing the location of needle injection (upper panel) and expression of the lentiviral vector (lower panel) in the CA1 area. *n* = 3. Scale bar equals 50 μm. **B** Different Lenti-EGFP-tagged vectors were transducted to the hippocampus of WT mice or APP/PS mice and their spatial learning performance was shown 14 days after lentiviral vector transduction (*n* = 7) (F_3,24_ = 26.1, *P* < 0.001; q = 9.6, *P* < 0.001 comparing the Lenti-EGFP vector in APP/PS1 mice with Lenti-EGFP vector in WT mice; q = 5.06, *P* < 0.01 comparing the Lenti-EGFP-SUMO1 in APP/PS1 mice with Lenti-EGFP vector in APP/PS1 mice; q = 6.72, *P* < 0.001 comparing the Lenti-EGFP-SUMO1 in APP/PS1 mice with Lenti-EGFP-SUMO1ΔGG in APP/PS1 mice). **C** Probe trial performance showing the time spent in each of the four quadrants from the same mice (*n* = 7) (F_3,24_ = 26.78, *P* < 0.001; q = 10.71, *P* < 0.001 comparing the Lenti-EGFP-vector in APP/PS1 mice with Lenti-EGFP-vector in WT mice; q = 6.0, *P* < 0.001 comparing the Lenti-EGFP-SUMO1 in APP/PS1 mice with Lenti-EGFP-vector in APP/PS1 mice; q = 5.93, *P* < 0.001 comparing the Lenti-EGFP-SUMO1ΔGG in APP/PS1 mice with Lenti-EGFP-SUMO1 in APP/PS1 mice). **D** Probe trial performance showing the distance travelled in the target quadrant from the same animals (*n* = 7) (left panel) (F_3,24_ = 20.57, *P* < 0.001; q = 9.99, *P* < 0.001 comparing the Lenti-EGFP vector in APP/PS1 mice with Lenti-EGFP vector in WT mice; q = 4.93, *P* < 0.01 comparing the Lenti-EGFP-SUMO1 in APP/PS1 mice with Lenti-EGFP vector in APP/PS1 mice; q = 3.92, *P* < 0.05 comparing the Lenti-EGFP-SUMO1ΔGG in APP/PS1 mice with Lenti-EGFP-SUMO1 in APP/PS1 mice). Probe trial performance showing platform latency from the same animals (right panel) (F_3,24_ = 4.73, *P* = 0.01; q = 3.63, *P* < 0.05 comparing the Lenti-EGFP vector in APP/PS1 mice with Lenti-EGFP vector in WT mice; q = 3.1, *P* < 0.05 comparing the Lenti-EGFP-SUMO1 in APP/PS1 mice with Lenti-EGFP vector in APP/PS1 mice; q = 3.79, *P* < 0.05 comparing the Lenti-EGFP-SUMO1ΔGG in APP/PS1 mice with Lenti-EGFP-SUMO1 in APP/PS1 mice). **E** Recognition training and recognition memory performance from the same animals is shown (*n* = 7). Time spent exploring object C and object A during the recognition memory test for WT mice receiving Lenti-EGFP vector transduction (t_1,12_ = 17.03, *P* < 0.001). Time spent exploring object C and object A during the recognition memory test for APP/PS1 mice receiving Lenti-EGFP-SUMO1 transduction (t_1,12_ = 6.81, *P* < 0.001). Data are mean ± SEM. * *P* < 0.05, ** *P* = 0.01, ^**#**^ *P* < 0.001

The above results together revealed that SUMOylation of APP decreased the association between APP and BACE1, decreased the expression of BACE1 and sAPPβ and increased the degradation of BACE1. It also increased the expression of sAPPα. In this study, we examined the physiological significance of APP SUMOylation. The water maze learning task was adopted to study this issue. Animals were divided to four groups and received different Lenti-EGFP-tagged vector transductions as that described in Fig. 2C. They were subjected to water maze learning 14 days later. The location of Lenti-EGFP vector transduction and expression is shown in Fig. 4A. Results revealed that APP/PS1 mice receiving Lenti-EGFP vector transduction showed impaired acquisition performance compared with WT mice receiving Lenti-EGFP vector transduction. But transduction of Lenti-EGFP-SUMO1 vector to APP/PS1 mice rescues the acquisition deficit, whereas transduction of Lenti-EGFP-SUMO1△GG vector to APP/PS1 mice worsened the acquisition performance (Fig. 4B). Their retention performance is similar to their acquisition performance. APP/PS1 mice receiving Lenti-EGFP vector transduction showed impaired memory retention compared with WT mice receiving Lenti-EGFP vector transduction. Transduction of Lenti-EGFP-SUMO1 vector to APP/PS1 mice rescues the retention deficit, but transduction of Lenti-EGFP-SUMO1△GG vector to APP/PS1 mice yielded a similar retention performance as that of APP/PS1 mice receiving Lenti-EGFP vector transduction (Fig. 4C). The total distance travelled in the target region and platform latency for the probe trial test of these animals parallel with their retention performance and are shown in Fig. 4D, left panel and right panel, respectively. But the swim speed of these four groups of mice is similar (Supplementary Fig. 3 A). These animals were also subjected to visible platform learning three days after the retention test and their visible platform learning performance is shown in Supplementary Fig. 3B.

### Lenti-EGFP-SUMO1 transduction to APP/PS1 mice rescues recognition memory deficit compared with APP/PS1 mice receiving Lenti-EGFP vector transduction

The same animals subjected to the above spatial learning and memory test were also subjected to recognition training and memory test one week after visible platform learning. Results revealed that animals in all four groups performed similarly in exploring object A and object B during the training period. During the memory recognition test, WT mice apparently spent more time exploring object C than object A. APP/PS1 mice receiving Lenti-EGFP-vector transduction spent similar amount of time exploring object C and object A, but APP/PS1 mice receiving Lenti-EGFP-SUMO1 transduction has reversed the situation and they spent more time exploring object C than object A. However, APP/PS1 mice receiving Lenti-EGFP-SUMO1GG transduction seem to not recognize the novel object C and they even spent relatively more time exploring object A than object C (Fig. 4E).

### APP/PS1 mice receiving Lenti-EGFP-SUMO1 transduction show decreased Aβ and Aβ oligomerization and decreased amount of amyloid plaque compared with APP/PS1 mice receiving Lenti-EGFP vector transduction

**Fig. 5:**
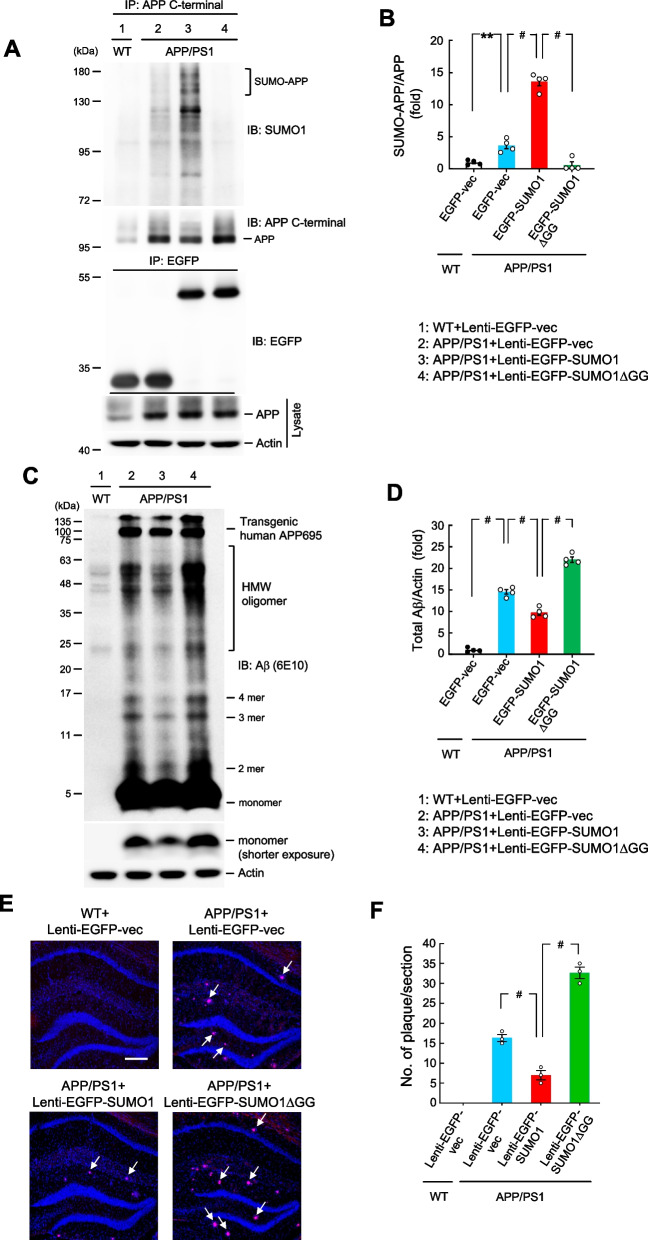
APP/PS1 mice receiving Lenti-EGFP-SUMO1 transduction show decreased Aβ and Aβ oligomerization and decreased amount of amyloid plaque compared with APP/PS1 mice receiving Lenti-EGFP vector transduction**. A** Four animals from each group were sacrificed after the recognition memory test and their hippocampal tissue was subjected to SUMOylation assay for determination of endogenous APP SUMOylation level. A representative gel pattern is shown (upper panel). The cell lysates were also immunoprecipitated and immunoblotted with anti-EGFP antibody for verification of Lenti-EGFP vector transduction and expression (middle panel). The amount of input APP is also shown (lower panel) (*n* = 4). **B** The quantified result of APP SUMOylation (*n* = 4) (F_3,12_ = 153.11, *P* < 0.001; q = 5.41, *P* < 0.01 comparing the Lenti-EGFP vector in APP/PS1 mice with Lenti-EGFP vector in WT mice; q = 20.27, *P* < 0.001 comparing the Lenti-EGFP-SUMO1 in APP/PS1 mice with Lenti-EGFP vector in APP/PS1 mice; q = 26.44, *P* < 0.001 comparing the Lenti-EGFP-SUMO1ΔGG in APP/PS1 mice with Lenti-EGFP-SUMO1 in APP/PS1 mice). **C** The same cell lysates were also subjected to western blot determination of Aβ and Aβ oligomerization expression (*n* = 4). **D** The quantified result of Aβ expression (F_3,12_ = 275.56, *P* < 0.001; q = 39.6, *P* < 0.001 comparing the Lenti-EGFP vector in APP/PS1 mice with Lenti-EGFP vector in WT mice; q = 9.1, *P* < 0.001 comparing the Lenti-EGFP-SUMO1 in APP/PS1 mice with Lenti-EGFP vector in APP/PS1 mice; q = 23.25, *P* < 0.001 comparing the Lenti-EGFP-SUMO1ΔGG in APP/PS1 mice with Lenti-EGFP-SUMO1 in APP/PS1 mice). **E** Proteostat dye staining showing amyloid plaque deposits (red) in the same groups of mice (*n* = 3 each group). Scale bar equals 200 μm. **F** The quantified result of amyloid plaque (F_3,8_ = 189.12, *P* < 0.001; q = 9.08, *P* < 0.001 comparing the Lenti-EGFP-SUMO1 in APP/PS1 mice with Lenti-EGFP vector in APP/PS1 mice; q = 24.98, *P* < 0.001 comparing the Lenti-EGFP-SUMO1ΔGG in APP/PS1 mice with Lenti-EGFP-SUMO1 in APP/PS1 mice). Data are mean ± SEM. ** *P* < 0.01, ^**#**^ *P* < 0.001

Animals were sacrificed after the recognition memory test and the hippocampal tissue from four animals of each group was subjected to SUMOylation assay for determination of APP protein SUMOylation. Results revealed that increased APP SUMOylation is apparently observed in APP/PS1 mice receiving Lenti-EGFP-SUMO1 transduction compared with that of three other groups (Fig. 5A). The quantified result of APP SUMOylation is shown in Fig. 5B. Western blot for Aβ expression was also carried out using the same tissue lysates. Results indicated that a fair amount of Aβ and Aβ oligomerization was observed in APP/PS1 mice receiving Lenti-EGFP vector transduction. Amyloid-beta and Aβ oligomerization level is apparently reduced in APP/PS1 mice receiving Lenti-EGFP-SUMO1 transduction, but they are dramatically increased in APP/PS1 mice receiving Lenti-EGFP-SUMO1ΔGG transduction (Fig. 5C). As commented for Fig. 2C, the 6E10 antibody also recognizes C99, and the C99 band is possibly confounded with the 3-mer Aβ band at ~ 11 kDa in Fig. 5C, but not the Aβ monomer and other Aβ oligomer bands (Supplementary Fig. 2 A). Therefore, it should not affect the result of total Aβ expression. The quantified result of total Aβ expression is shown in Fig. 5D.

Three animals from each of these four groups were subjected to immunohistochemistry staining of amyloid plaque using the ProteoStat Detection Kit. Results showed that there is almost no amyloid plaque in WT animals receiving Lenti-EGFP vector transduction. Some amyloid plaque is observed in APP/PS1 mice receiving Lenti-EGFP vector transduction, as indicated by the arrows. Transduction of Lenti-EGFP-SUMO1 to APP/PS1 mice apparently decreased the amount of amyloid plaque, whereas transduction of Lenti-EGFP-SUMO1ΔGG to APP/PS1 mice increased the amount of amyloid plaque compared with APP/PS1 mice receiving Lenti-EGFP vector transduction (Fig. 5E). To examine whether there may be neuronal loss in APP/PS1 mice receiving Lenti-EGFP-SUMO1ΔGG transduction, we have also measured the fluorescence intensity of DAPI staining in the dentate gyrus area from the same animals. The result showed that DAPI fluorescence intensity is slightly lower in APP/PS1 mice receiving Lenti-EGFP-vector transduction and in APP/PS1 mice receiving Lenti-EGFP-SUMO1ΔGG transduction compared with WT mice receiving Lenti-EGFP vector transduction, but the difference did not reach a statistically significant level (Supplementary Fig. 4). The quantified result of amyloid plaque is shown in Fig. 5F. We further examined whether the ProteoStat dye staining indeed stains amyloid plaque specifically. We have conducted additional immunohistochemistry using both the ProteoStat dye and anti-Aβ antibody using hippocampal tissue slices from APP/PS1 mice. The merged image showed that the ProteoStat dye staining and Aβ staining are well co-localized in the hippocampus (Supplementary Fig. 1).

### Melatonin increases Ubc9 expression and enhances APP SUMOylation

**Fig. 6:**
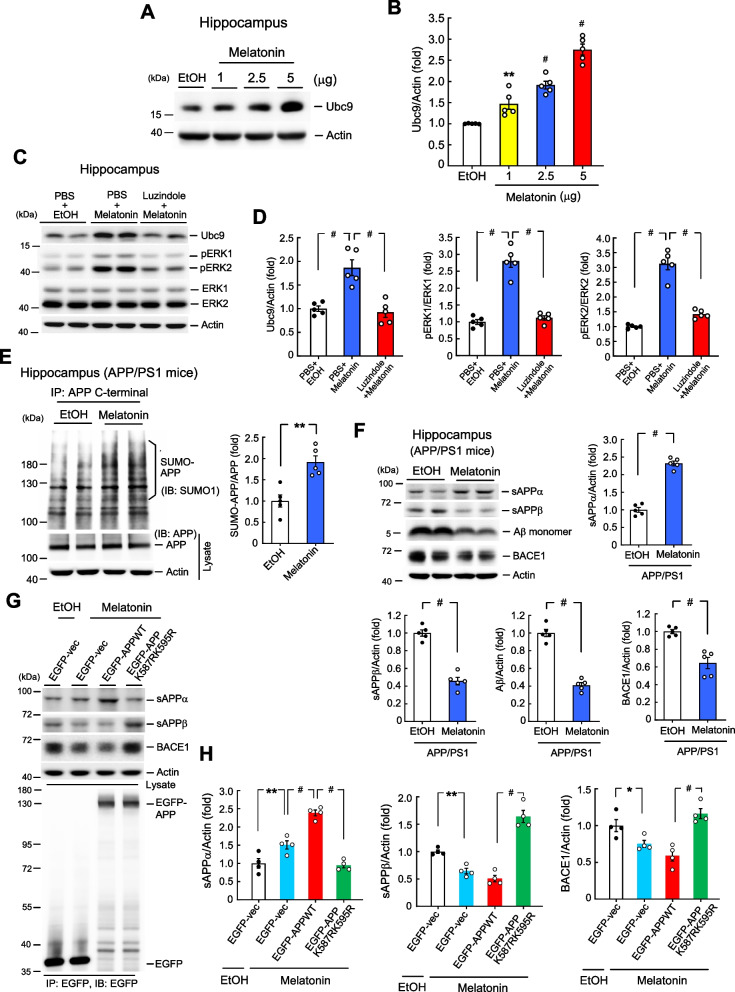
Melatonin increases Ubc9 expression and enhances APP SUMOylation.** A** Mice received different concentrations (1, 2.5 or 5 μg) of intra-hippocampal melatonin injection and their hippocampal tissue lysate was dissected out and subjected to western blot determination of Ubc9 expression 1 h later (*n* = 5). **B** The quantified result of Ubc9 expression (F_3,16_ = 53.17, *P* < 0.001; q = 4.58, *P* < 0.01 comparing the melatonin 2 μg group with EtOH group; q = 8.93, *P* < 0.001 comparing the melatonin 5 μg group with EtOH group; q = 17.13, *P* < 0.001 comparing the melatonin 10 μg group with EtOH group). **C** Mice received PBS + EtOH, PBS + melatonin (2.5 μg) or luzindole (0.4 μg) + melatonin (2.5 μg) injections to their hippocampus. The time interval between the first and second injection is 45 min. Animals were sacrificed 1 h after the second injection and their hippocampal tissue is subjected to western blot determination of Ubc9, pERK1/2 and ERK1/2 expression (*n* = 5). **D** The quantified result of Ubc9 expression (left panel) (F_2,12_ = 19.41, *P* < 0.001; q = 7.29, *P* < 0.001 comparing the PBS + melatonin group with PBS + EtOH group; q = 7.93, *P* < 0.001 comparing the luzindole + melatonin group with PBS + melatonin group); and the quantified result of pERK1 expression (middle panel) (F_2,12_ = 67.73, *P* < 0.001; q = 14.71, *P* < 0.001 comparing the PBS + melatonin group with PBS + EtOH group; q = 13.75, *P* < 0.001 comparing the luzindole + melatonin group with PBS + melatonin group); and the quantified result of pERK2 expression (right panel) (F_2,12_ = 80.78, *P* < 0.001; q = 16.97, *P* < 0.001 comparing the PBS + melatonin group with PBS + EtOH group; q = 13.61, *P* < 0.001 comparing the luzindole + melatonin group with PBS + melatonin group). **E** EtOH (20%) or melatonin (30 μl, 10 μg/μl concentration) was injected to APP/PS1 mice (i.p.) at one injection per day for 21 days consecutively and their hippocampal tissue was dissected out for determination of APP SUMOylation level 3 days after the last injection. The amount of input APP is also shown (left panel) (*n* = 5). The quantified result is shown in the right panel (t_1,8_ = 4.43, *P* < 0.01). **F** The same tissue lysate from (**E**) was also subjected to western blot determination of sAPPα, sAPPβ, Aβ and BACE1 expression (upper-left panel) (*n* = 5). The quantified result of sAPPα expression (upper-right panel) (t_1,8_ = 13.62, *P* < 0.001), sAPPβ expression (lower-left panel) (t_1,8_ = 9.81, *P* < 0.001), Aβ expression (lower-middle panel) (t_1,8_ = 11.55, *P* < 0.001) and BACE1 expression (lower-right panel) (t_1,8_ = 5.3, *P* < 0.001). **G** WT mice received intra-hippocampal EGFP-vector + EtOH, EGFP-vector + melatonin (2.5 μg), EGFP-APPWT + melatonin (2.5 μg) or EGFP-APPK587RK595R + melatonin (2.5 μg) transfection and injection. EtOH or melatonin was administered 47 h after plasmid transfection and animals were sacrificed 1 h after EtOH/melatonin injection. Their hippocampal tissue was subjected to western blot determination of sAPPα, sAPPβ and BACE1 expression. Cell lysate was also immunoprecipitated and immunoblotted with anti-EGFP antibody to confirm the transfection and expression of the plasmid (lower panel) (*n* = 4). **H** The quantified result of sAPPα expression (left panel) (F_3,12_ = 4.93, *P* < 0.001; q = 4.94, *P* < 0.01 comparing the EGFP-vector + melatonin group with EGFP-vector + EtOH group; q = 8.52, *P* < 0.001 comparing the EGFP-APPWT + melatonin group with EGFP-vector + melatonin group; q = 13.97, *P* < 0.001 comparing the EGFP-APPK587RK595R + melatonin group with EGFP-APPWT + melatonin group). The quantified result of sAPPβ expression (middle panel) (F_3,12_ = 63.48, *P* < 0.001; q = 4.06, *P* = 0.01 comparing the EGFP-vector + melatonin group with EGFP-vector + EtOH group; q = 17.59, *P* < 0.001 comparing the EGFP-APPK587RK595R + melatonin group with EGFP-APPWT + melatonin group). The quantified result of BACE1 expression (F_3,12_ = 13.53, *P* < 0.001; q = 3.59, *P* < 0.05 comparing the EGFP-vector + melatonin group with EGFP-vector + EtOH group; q = 8.26, *P* < 0.001 comparing the EGFP-APPK587RK595R + melatonin group with EGFP-APPWT + melatonin group). Data are mean ± SEM. * *P* < 0.05, ** *P* ≤ 0.01, ^**#**^ *P* < 0.001

The above results demonstrated that APP can be SUMO-modified by Ubc9 in the hippocampus. Next, we aimed to identify an endogenous stimulus that regulates APP SUMOylation. Melatonin is a pineal hormone and melatonin levels are shown to be lower in AD patients than in age-matched controls (Wu et al. 2003). Further, the progression of AD pathology was shown to parallel with a decline in cerebrospinal fluid melatonin levels (Zhou et al. 2003). Moreover, results from transgenic animal studies suggest that melatonin alleviates the pathology of AD and increases survival (Matsubara et al. 2003; Shukla et al. 2017). Here, we examined whether melatonin produces a protective effect against AD through enhanced SUMOylation of APP. Because we have shown that APP is SUMO-modified by Ubc9, we first examined whether melatonin increases the expression level of Ubc9. Mice were divided to four groups and received intra-hippocampal injection of ethanol or different concentrations of melatonin. Animals were sacrificed 1 h after injection and their hippocampal tissue is subjected to western blot determination of Ubc9 expression. Results indicated that acute melatonin produced a dose-dependent increase of Ubc9 expression (Fig. 6A and B). We next examined whether the effect of melatonin on Ubc9 expression is mediated through the melatonin receptors. The melatonin receptor antagonist luzindole was used. Mice were divided to three groups and received PBS + EtOH, PBS + melatonin or luzindole + melatonin injections. The time interval between the first and second injections is 45 min. Animals were sacrificed 1 h after the second injection and their hippocampal tissue is subjected to western blot determination of Ubc9 expression. Because melatonin receptor activation is known to activate the MAPK pathway (Shukla et al. 2017), the same tissue lysates were also subjected to western blot determination of pERK1/2 level and ERK1/2 level to confirm the effectiveness of melatonin injection. Results showed that melatonin consistently increased Ubc9 expression, but this effect was abolished by prior luzindole injection. Melatonin also increased the phosphorylation level of ERK1/2, and this effect was similarly blocked by prior luzindole injection. But these treatments did not affect ERK1/2 expression levels (Fig. 6C and D).

We next examined whether melatonin alters the APP SUMOylation level in APP/PS1 mice. Mice were divided to two groups and received intraperitoneal EtOH or melatonin injection for 21 days consecutively. They were sacrificed 3 days after the last injection and their hippocampal tissue was subjected to the SUMOylation assay. Result revealed that sub-chronic melatonin injection significantly increased the SUMOylation level of the APP protein (Fig. 6E). We have shown above that APP SUMOylation promotes the nonamyloidogenic pathway, therefore, we expected that melatonin should increase the expression level of sAPPα, and decrease the expression level of sAPPβ, Aβ and BACE1. This issue was examined here and the same tissue lysates from APP SUMOylation assay were used. Results indicated that sub-chronic melatonin injection significantly increased sAPPαexpression, but it decreased sAPPβ, Aβ and BACE1 expression (Fig. 6F). In order to validate these results, we have used another sAPPα antibody (from IBL) to carry out western blot with the sample lysate. Result indicated that melatonin consistently increased sAPPα expression (Supplementary Fig. 5 A). We also used the 22C11 antibody to conduct western blot with the same lysate and the result consistently showed that melatonin markedly decreased sAPPβ expression (Supplementary Fig. 5B). We lastly examined whether the altered expression level of sAPPα, sAPPβ, Aβ and BACE1 by melatonin is indeed due to melatonin induction of APP SUMOylation. Wild-type mice were divided to four groups and received intra-hippocampal EGFP-vector + EtOH, EGFP-vector + melatonin, EGFP-APPWT + melatonin or EGFP-APPK587RK595R + melatonin plasmid transfection and injection. EtOH or melatonin was administered 47 h after plasmid transfection and animals were sacrificed 1 h after EtOH/melatonin injection. Their hippocampal tissue was subjected to western blot determination of sAPPα, sAPPβ and BACE1 expression. Results revealed that melatonin increased sAPPα expression compared with EtOH injection. Melatonin further increased sAPPα expression in mice transfected with EGFP-APPWT plasmid compared with mice transfected with EGFP-vector due to overexpression of the APP protein. But melatonin decreased sAPPα expression in mice transfected with the EGFP-APP sumo-mutant plasmid compared with mice transfected with EGFP-APPWT plasmid. The effects of melatonin and EGFP-tagged plasmid transfection on sAPPβ and BACE1 expression are opposite to that of sAPPα expression (Fig. 6G). The quantified results are shown in Fig. 6H. The tissue lysates were also immunoprecipitated with anti-EGFP antibody and immunoblotted with anti-EGFP antibody to confirm the transfection and expression of EGFP-tagged plasmids (Fig. 6G, lower panel). Similarly, to validate these results, we have used another sAPPα antibody (from IBL) and the 22C11 antibody to carry out western blot with the same lysate. Results revealed similar expression pattern for both sAPPα (Supplementary Fig. 5 C) and sAPPβ (Supplementary Fig. 5D) as that shown in Fig. 6G.

### Blockade of APP phosphorylation promotes APP SUMOylation

**Fig. 7:**
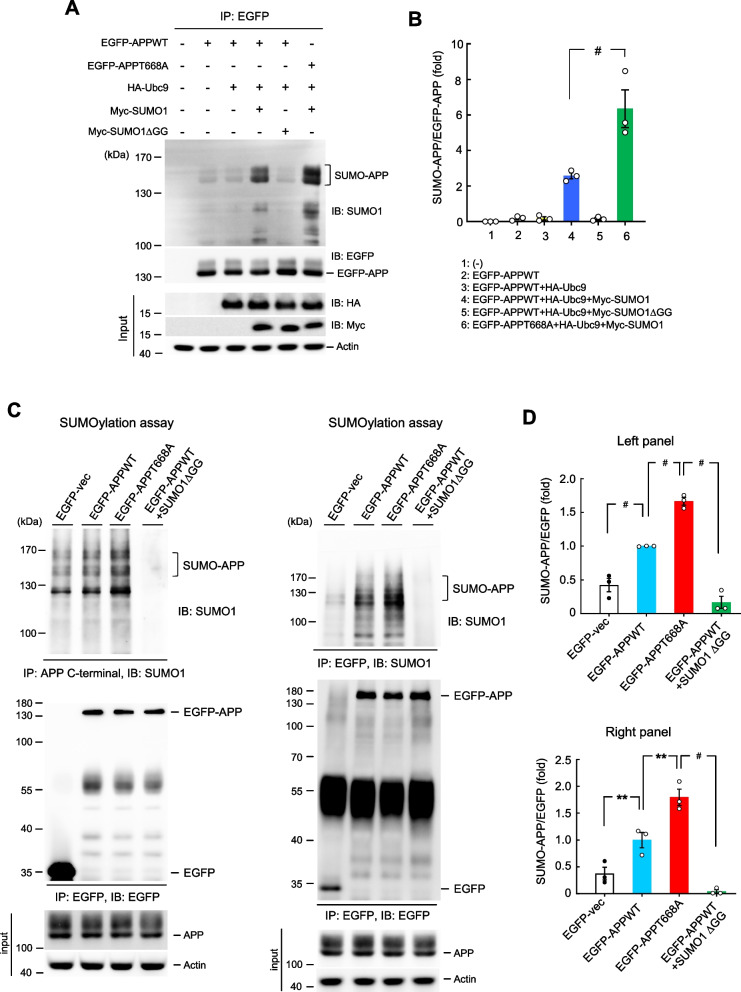
Blockade of APP phosphorylation promotes APP SUMOylation. **A** EGFP-APPWT or EGFP-APPT668A phosphorylation mutant plasmid was co-transfected with HA-Ubc9 and Myc-SUMO1 or Myc-SUMO1ΔGG plasmid to HEK293T cells. Cell lysates were immunoprecipitated with anti-EGFP antibody and immunoblotted with anti-SUMO1 antibody for determination of the APP SUMOylation level 48 h later. Immunoblotting with antibodies against various tags was carried out to verify the transfection and expression of various plasmids. Experiments are in three repeats. **B** The quantified result of APP SUMOylation (F_5,12_ = 32.39, *P* < 0.001; q = 8.48, *P* < 0.001 comparing the EGFP-APPT668A + HA-Ubc9 + Myc-SUMO1 group with EGFP-APPWT + HA-Ubc9 + Myc-SUMO1 group). **C** WT mice received EGFP-vector, EGFP-APPWT or EGFP-APPT668A plasmid transfection. For another group, animals received EGFP-APPWT transfection but with the SUMO1ΔGG mutant protein added to the reaction. Their hippocampal tissue was dissected out and subjected to APP SUMOylation assay 48 h later. Tissue lysates were immunoprecipitated with anti-APP C-terminal antibody and immunoblotted with anti-SUMO1 antibody (upper-left panel) (*n* = 3). The same tissue lysates were also immunoprecipitated and immunoblotted with anti-EGFP antibody for verification of plasmid transfection and expression (middle-left panel) (*n* = 3). The amount of input APP is also shown (lower-left panel) (*n* = 3). Tissue lysates from a different batch of animals receiving the same plasmid transfection as that in (**C**) were also subjected to APP SUMOylation assay. Lysates were immunoprecipitated with anti-EGFP antibody and immunoblotted with anti-SUMO1 antibody (upper-right panel) (*n* = 3). The same tissue lysates were also immunoprecipitated and immunoblotted with anti-EGFP antibody for verification of plasmid transfection and expression (middle-right panel) (*n* = 3). The amount of input APP is also shown (lower-right panel) (*n* = 3). **D** The quantified result of APP SUMOylation from left panel of (**C**) (upper panel) (F_3,8_ = 83.72, *P* < 0.001; q = 7.94, *P* < 0.001 comparing the EGFP-APPWT group with EGFP-vector group; q = 9.15, *P* < 0.001 comparing the EGFP-APPT668A group with EGFP-APPWT group; q = 20.57, *P* < 0.001 comparing the EGFP-APPWT + SUMO1ΔGG group with EGFP-APPT668A group). The quantified result of APP SUMOylation from right panel of (C) (lower panel) (F_3,8_ = 40.65, *P* < 0.001; q = 5.17, *P* < 0.01 comparing the EGFP-APPWT group with EGFP-vector group; q = 6.59, *P* < 0.01 comparing the EGFP-APPT668A group with EGFP-APPWT group; q = 14.48, *P* < 0.001 comparing the EGFP-APPWT + SUMO1ΔGG group with EGFP-APPT668A group). Data are mean ± SEM. ^**#**^ *P* < 0.001

APP is shown to be phosphorylated at Thr-668 and APP Thr-668 phosphorylation level was found significantly increased in the hippocampus of AD patients (Lee et al. 2003a, b). Further, death-associated protein kinase 1 (DAPK1) is highly expressed in the AD brain and inhibition of DAPK1 was shown to decrease APP phosphorylation at Thr-668 and attenuate amyloidogenic processing of APP (Kim et al. 2016). Here, we examined whether APP phosphorylation at Thr-668 may affect the level of APP SUMOylation. EGFP-APPWT or EGFP-APPT668A phosphorylation mutant plasmid was co-transfected with HA-Ubc9 and Myc-SUMO1 or Myc-SUMO1ΔGG plasmid to HEK293T cells. Forty-eight hours later, the cell lysates were immunoprecipitated with anti-EGFP antibody and immunoblotted with anti-SUMO1 antibody for determination of the APP SUMOylation level. Immunoblotting with antibodies against various tags was carried out to verify the transfection and expression of various plasmids. Results indicated that APP SUMOylation was apparently observed when EGFP-APPWT plasmid was co-transfected with HA-Ubc9 and Myc-SUMO1 plasmids compared with EGFP-APPWT and HA-Ubc9 plasmid transfections. This effect was completely abolished when Myc-SUMO1ΔGG, instead of Myc-SUMO1, was co-transfected. However, APP SUMOylation level was further increased when EGFP-APPT668A phosphorylation mutant, instead of EGFP-APPWT, was transfected (Fig. 7A). The quantified result is shown in Fig. 7B. We are also interested to know whether transfection of EGFP-APPT668D, the phosphorylation-mimicking mutant, produced an effect opposite to that of EGFP-APPT668A. HEK293T cells were transfected with the same plasmids as described in Fig. 7A except that the EGFP-APPT668A plasmid was replaced with the EGFP-APPT668D plasmid. Result indicated that transfection of EGFP-APPT668D significantly decreased APP SUMOylation compared with EGFP-APPWT transfection (Supplementary Fig. 6 A). The quantified result is shown in Supplementary Fig. 6B.

We next examined whether APP phosphorylation at Thr-668 also affects APP SUMOylation in the hippocampus. Wild-type mice were divided to four groups. Animals received EGFP-vector, EGFP-APPWT or EGFP-APPT668A plasmid transfection. For one of these groups, animals received EGFP-APPWT transfection but with the SUMO1ΔGG mutant protein added to the reaction. Their hippocampal tissue was subjected to APP SUMOylation assay 48 h later. Results showed that transfection of EGFP-APPWT increases the level of APP SUMOylation compared with EGFP-vector transfection. Transfection of EGFP-APPT668A further increased the level of APP SUMOylation compared with EGFP-APPWT transfection. But APP SUMOylation was completely abolished when SUMO1ΔGG mutant protein was added to the reaction of the EGFP-APPWT transfection group (Fig. 7C, upper-left panel). The quantified result is shown in Fig. 7D, upper panel. Plasmid transfection and expression was confirmed by immunoprecipitation and immunoblotting with anti-EGFP antibody (Fig. 7C, middle-left panel). The amount of input APP is also shown (Fig. 7C, lower-left panel). In this experiment, the SUMO-APP bands were observed in all three groups of animals. To confirm the expression and alteration of APP SUMOylation by these treatments, a different batch of animals receiving the same plasmid transfection were adopted. Their tissue lysates were immunoprecipitated with anti-EGFP antibody and immunoblotted with anti-SUMO1 antibody. Similar results were obtained. APP SUMOylation was barely observed in animals receiving EGFP-vector transfection. Transfection of EGFP-APPWT increases the level of APP SUMOylation compared with EGFP-vector transfection. Transfection of EGFP-APPT668A further increased the level of APP SUMOylation compared with EGFP-APPWT transfection. But APP SUMOylation was completely abolished when SUMO1ΔGG mutant protein was added to the reaction of the EGFP-APPWT transfection group (Fig. 7C, upper-right panel). The quantified result is shown in Fig. 7D, lower panel. Plasmid transfection and expression was confirmed by immunoprecipitation and immunoblotting with anti-EGFP antibody (Fig. 7C, middle-right panel). The amount of input APP is also shown (Fig. 7C, lower-right panel).

The above results revealed that APP Thr-668 phosphorylation negatively regulates APP SUMOylation. We have also shown that melatonin increased APP SUMOylation. Based on these results, it is expected that melatonin may decrease the phosphorylation level of APP. This speculation was examined here. Animals were divided to the following three groups: WT mice received sub-chronic EtOH injection (i.p.), APP/PS1 mice received sub-chronic EtOH injection (i.p.) and APP/PS1 mice received sub-chronic (i.p.) melatonin injection for 21 days. Animals were sacrificed 3 days after the last injection and their hippocampal tissue was subjected to western blot determination of p-Thr668 APP phosphorylation, transgenic human APP695 expression and total APP protein expression. Results showed that p-Thr668 APP phosphorylation level is lower in APP/PS1 mice receiving EtOH injection compared to WT mice receiving EtOH injection. But APP Thr-668 phosphorylation level was significantly decreased in APP/PS1 mice receiving melatonin injection compared to APP/PS1 mice receiving EtOH injection (Supplementary Fig. 7 A). The quantified result is shown in Supplementary Fig. 7B. Immunoblotting with anti-6E10 antibody revealed that the transgenic human APP695 protein is expressed in both groups of APP/PS1 mice (Supplementary Fig. 7 A, lower panel).

## Discussion

The present results demonstrated that APP is SUMO-modified by Ubc9 in the mouse hippocampus at Lys-587 and Lys-595. APP SUMOylation prevents BACE1 association with APP and cleavage of APP and enhances BACE1 degradation. Transduction of the Lenti-EGFP-SUMO1 vector to the hippocampus of APP/PS1 mice reduces the amount of Aβ and amyloid plaque, decreases the expression of sAPPβ and BACE1, but increases the expression of sAPPα. Lenti-EGFP-SUMO1 transduction also rescues spatial memory and recognition memory deficits in APP/PS1 mice. Transduction of Lenti-EGFP-SUMO1ΔGG to APP/PS1 mice produces an opposite effect for the above measures. These results together suggest that APP SUMOylation promotes the non-amyloidogenic pathway and produces a protective effect against Aβ toxicity. Further, we identified melatonin as an endogenous stimulus that enhances APP SUMOylation and promotes non-amyloidogenic processing of APP.

These results are consistent with a previous report showing that APP is SUMO-modified at Lys-587 and Lys-595 in HeLa cells, and APP SUMOylation decreases Aβ aggregates in cells transfected with mutant APP (Zhang and Sarge 2008)**.** But we have extended this issue and examined the role and mechanism as well as the physiological significance of APP SUMOylation in animals. In this study, we have demonstrated that APP SUMOylation takes place in the brain of APP/PS1 mice endogenously when EGFP-SUMO1 plasmid was transfected to the hippocampus of these animals. We also demonstrated that APP is SUMO-modified at the same residues, Lys-587 and Lys-595, as that in the cell. However, our result also indicated that transfection of the K587RK595R double mutant, although significantly decreased, but did not completely abolish APP SUMOylation, compared with that of SUMO1ΔGG mutant protein addition (Fig. 1D). This result implicates that there might be other residue(s) on the APP protein that could also be SUMO-modified. Further studies are required to clarify this issue. Our results have shown that transduction of the Lenti-EGFP-SUMO1 vector to APP/PS1 mice decreased total Aβ level compared with Lenti-EGFP-vector transduction to APP/PS1 mice. However, in Fig. 2C, expression of SUMO1 apparently decreased Aβ monomer and its higher oligomers, but not Aβ trimers and tetramers. This is probably because at the time of tissue dissection, cells are at the stage of starting of transition from trimers and tetramers to higher oligomers, therefore, more trimers and tetramers were observed but significantly less higher oligomers were seen. However, this is not always the situation because in another experiment with APP/PS1 mice receiving the same transduction, SUMO1 expression markedly decreased Aβ monomer, trimers, tetramers and higher oligomers compared with animals receiving Lenti-EGFP-vector transduction (Fig. 5C). For the behavioral experiments we have shown that transduction of Lenti-EGFP-SUMO1 vector to APP/PS1 mice rescues spatial memory and recognition memory deficits, and the APP SUMOylation level is increased in these animals. But we did not provide the direct evidence that these effects are indeed due to APP SUMOylation. The reason is because that the Lenti-EGFP-APPK587RK595R vector is too large to be successfully constructed and transducted to the mouse brain. We have previously shown that the APP C-terminal fragment protein AICD could also be SUMO-modified (Liu et al. 2021). Although we can not exclude the possibility that AICD SUMOylation is also increased in APP/PS1 mice transducted with the Lenti-EGFP-SUMO1 vector, it is unlikely that AICD SUMOylation contributes to decreased association between APP and BACE1 because AICD SUMOylation takes place in the nucleus whereas the interaction between APP and BACE1 mainly occurs in the endosome. In addition, we have found that melatonin increases the level of endogenous APP SUMOylation and promotes the nonamyloidogenic pathway, and that blockade of APP SUMOylation prevents this effect of melatonin. However, other possibilities may also exist. For example, we have previously shown that melatonin also induces endogenous AICD SUMOylation and AICD SUMOylation similarly protects against Aβ toxicity. Melatonin may also protect against Aβ toxicity through altered SUMOylation of other proteins. In this study, melatonin was found to increase Ubc9 expression rapidly in 1 h. This is probably because that melatonin was directly injected to the mouse hippocampus and it rapidly activates melatonin receptor and its downstream signaling. This explanation is supported by our finding that melatonin injection dramatically induces MAPK/ERK activation. This explanation is also consistent with another report showing that melatonin receptor activation activates the MAPK pathway (Shukla et al. 2017). Similar result was found in our previous study that direct injection of melatonin to the rat hippocampus significantly increases the expression of PIAS1, a SUMO E3 ligase, in 1 h (Liu et al. 2021).

Our results revealed that SUMOylation of APP facilitates the degradation of BACE1. Because we have shown that APP SUMOylation prevents BACE1 association with APP, it is conceivable that the non-associated BACE1 is easier to be degraded. On the opposite, when APP SUMOylation is prevented by Myc-SUMO1ΔGG transfection, BACE1 degrades more slowly, and this is perhaps due to stronger BACE1 association with the APP protein. Further, we have shown that blockade of APP SUMOylation by APP sumo-mutant plasmid transfection not only increased the expression level of BACE1, but also decreased the expression level of sumo-mutant APP protein (Fig. 3C and D). The decreased expression of sumo-mutant APP is possibly due to de-stabilization of this protein resulted from blockade of APP SUMOylation. This speculation is consistent with the notion that protein SUMOylation stabilizes the proteins (Geiss-Friedlander and Melchior 2007). This result is also consistent with our previous findings that SUMOylation of Hes-1 and Akt stabilizes Hes-1 and Akt, respectively (Chiou et al. 2014; Lin et al. 2016).

In the present study, we have adopted the APP/PS1 transgenic mice as the animal model for AD, it is possible that the observed memory-rescuing effect and amyloid plaque-reducing effect of Lenti-EGFP-SUMO1 transduction to APP/PS1 mice could partly due to SUMOylation of PSEN1 (PS1) in addition to SUMOylation of APP. To test this possibility, a different batch of wild-type mice received intra-hippocampal EGFP-vector transduction, EGFP-PSEN1 plasmid transfection, EGFP-PSEN1 plasmid transfection with the SUMO1 protein added to the reaction or EGFP-PSEN1 plasmid transfection with the SUMO1ΔGG mutant protein added to the reaction. Animals were sacrificed 48 h after plasmid transfection. Their hippocampal tissue lysate was immunoprecipitated with anti-EGFP antibody and immunoblotted with anti-SUMO1 antibody. The result revealed that transfection of the EGFP-PSEN1 plasmid slightly increased the endogenous PSEN1 SUMOylation level, but addition of the SUMO1 protein further enhanced the PSEN1 SUMOylation level, whereas PSEN1 SUMOylation level was completely abolished when the SUMO1ΔGG mutant protein was added to the reaction (Supplementary Fig. 8). In further analysis of the SUMO consensus motif on PSEN1, it is predicted that one of the three motifs is located in Exon 9 (at the position of a.a.314) (Zhao et al. 2014), whereas Exon 9 is deleted in APP/PS1 mice; therefore, PSEN1 SUMOylation is probably not as evident in APP/PS1 mice as that shown in the figure. These results together suggest that PSEN1 SUMOylation may also contribute to the observed memory-rescuing effect and amyloid plaque-reducing effect of Lenti-EGFP-SUMO1 transduction to APP/PS1 mice, but it does not affect the results of decreased association between APP and BACE1 and increased BACE1 degradation upon APP SUMOylation. It also does not affect our conclusion that APP SUMOylation promotes the nonamyloidogenic pathway.

The APP Thr-668 phosphorylation level was found increased in the AD brain (Lee et al. 2003a, b). Our results reveal that blockade of APP phosphorylation at Thr-668 dramatically increases the SUMOylation level of APP, whereas the phospho-mimicking mutant Thr-668D markedly decreases the SUMOylation level of APP. This result suggests that APP phosphorylation acts as a negative regulator of APP SUMOylation. One explanation for this observation is possibly due to conformational change of the APP protein resulted from blockade of the APP phosphorylation residue. Similar results were found with other proteins, such as c-Fos, c-Jun, p53 and Elk-1 (Bossis and Melchior 2006). However, our result also showed that the APP phosphorylation level, relatively to total APP, was slightly decreased in APP/PS1 mice compared with WT mice. This result is inconsistent with a previous report showing that APP phosphorylation is increased in the hippocampus of AD patients (Lee et al. 2003a, b). We do not know the explanation for this discrepancy yet. It could be that the endogenous APP phosphorylation level is higher in mice than in humans because the latter study was conducted in human brain. On the other hand, phosphorylation could also act as a positive regulator of SUMOylation for certain proteins, such as MeCP2 and MEF2 (Tai et al. 2016; Bossis and Melchior 2006). Moreover, consistent with our observation that melatonin increased endogenous APP SUMOylation level in APP/PS1 mice, melatonin, as expected, decreased endogenous APP Thr-668 phosphorylation level in APP/PS1 mice.

In the present study, the size of sAPPβ shown in the figures is below 72 kD, which is smaller than expected (95–100 kD). To confirm it is indeed the sAPPβ band, we have used the same lysate used in Supplementary Fig. 2 A (immunoblotted with APP C-terminal antibody) to first repeat the same western blot using the APP C-terminal antibody. As shown in the upper panel of Supplementary Fig. 9, we obtained similar expression pattern for both APP-CTFbeta and APP-CTFalpha as that shown in Supplementary Fig. 2A. We then stripped the membrane and immunoblotted with the sAPPβ antibody. Result revealed that the sAPPβ band is still below 72 kD (Supplementary Fig. 9, lower panel) as that shown in Supplementary Fig. 2B when we immunoblotted with the 22C11 antibody. The alteration of sAPPβ expression among the four groups is also consistent with that of APP CTFbeta (both sAPPβ and APP CTFbeta are the products of β-secretase cleavage). This result indicated that the sAPPβ band is consistently below 72 kD when we immunoblotted with either the sAPPβ antibody or the 22C11 antibody. An explanation for the smaller kD of the sAPPβ band is possibly because that APP is cleaved by caspases into different fragments and one of the fragments is sAPPβ which is around 70 kD. This explanation is supported by the findings that APP is directly cleaved by caspases (LeBlanc et al. 1999) and inhibition of caspase activity in APP-overexpressed cells yields a few sAPPβ fragments with one fragment shows 72 kD in size (Jean-Louis et al. 2018). It is possible that the caspase activity is high in the brain so the major band of sAPPβ around 70 kD is observed in the present study. When we examined the expanded gel of Fig. 2D (in Supplementary Fig. 10, the corresponding original blot), a band around 100 kD was barely observed and its expression pattern is similar to that of sAPPβ shown below 72 kD. Other studies supporting this speculation have shown that caspase-6 activation is implicated in the pathophysiology of AD and caspase-6 activity is high in the cortex, and it is increased with aging (Albrecht et al. 2009; Lessard-Beaudoin et al. 2016).

In summary, we have presently found that the APP protein can be SUMO-modified at Lys-587 and Lys-595 in the mouse brain endogenously. SUMOylation of APP prevents BACE1 association with and cleavage of the APP protein and therefore promotes the nonamyloidogenic pathway. Melatonin is an endogenous stimulus that inhibits APP phosphorylation and increases APP SUMOylation to facilitate nonamyloidogenic processing of APP.

## Supplementary Information

Additional file 1. Figure S1. ProteoStat dye and Aβ staining co-localize in the hippocampus. The mouse hippocampal tissue slice was subjected to immunohistochemistry staining of ProteoStat dye and Aβ. The images of ProteoStat dye staining and Aβ staining and their merged image are shown. Scale bar equals 200 μm for both the upper panel and lower panel.

Additional file 2. Figure S2. APP SUMOylation regulates APP-CTFalpha, APP-CTFbeta and sAPPβ expression. Lenti-EGFP vector or Lenti-EGFP-SUMO1 or Lenti-EGFP-SUMO1∆GG plasmid was transducted to the hippocampus of WT or APP/PS1 mice. Their CA1 tissue was dissected out and the tissue lysate was subjected to western blot determination of APP-CTFalpha and APP-CTFbeta expression using anti-APP C-terminal antibody. The same tissue lysate was also subjected to western blot determination of sAPP expression using 22C11 antibody. HEK293T cells were transfected with EGFP-vector or EGFP-sAPPα plasmid. Cell lysates were subjected to immunoblotting with anti-sAPPα antibody to determine the expression of EGFP-sAPPα. Immunoblotting with anti-EGFP antibody was used to verify plasmid transfection and expression. HEK293T cells were transfected with EGFP-vector or EGFP-sAPP plasmid. Cell lysates were subjected to immunoblotting with anti-sAPP antibody to determine the expression of EGFP-sAPP. Immunoblotting with anti-EGFP antibody was used to verify plasmid transfection and expression.

Additional file 3. Figure S3. Lenti-EGFP vector, Lenti-EGFP-SUMO1 or Lenti-EGFP-SUMO1ΔGG transduction do not affect swim speed of probe trial test in APP/PS1 mice. Animals received intra-hippocampal transduction of Lenti-EGFP vector, Lenti-EGFP-SUMO1 vector or Lenti-EGFP-SUMO1ΔGG vector and their swim speeds for the probe trial test are shown. Visible platform performance from the same animals is shown. Data are mean±SEM.

Additional file 4. Figure S4. DAPI staining intensity is not altered in APP/PS1 mice receiving. Lenti-EGFP-vector, Lenti-EGFP-SUMO1 or Lenti-EGFP-SUMO1∆GG transduction to the hippocampus. WT mice received Lenti-EGFP-vector transduction and APP/PS1 mice received Lenti-EGFP-vector, Lenti-EGFP-SUMO1 or Lenti-EGFP-SUMO1∆GG transduction as described in Figure 5E. Immunohistochemistry of DAPI staining was performed and the fluorescence intensity of DAPI staining in the marked area was measured. Scale bar equals 200 μm. The quantified result of DAPI fluorescence intensity for each group. Data are expressed as mean±SEM.

Additional file 5. Figure S5. Melatonin increases sAPPα expression and decreases sAPPβ expression and these effects were blocked by APP sumo-mutant transfection Mice were divided to two groups and received intraperitoneal EtOH or melatonin injection for 21 days consecutively. They were sacrificed 3 days after the last injection and their hippocampal tissue was subjected to western blot determinations of sAPPα expression using a different sAPPα antibody and sAPPβ expression using 22C11 antibody. Wild-type mice were divided to four groups and received intra-hippocampal EGFP-vector+EtOH, EGFP-vector+melatonin, EGFP-APPWT+melatonin or EGFP-APPK587RK595R+melatonin plasmid transfection and injection. EtOH or melatonin was administered 47 h after plasmid transfection and animals were sacrificed 1 h after EtOH/melatonin injection. Their hippocampal tissue was subjected to western blot determination of sAPPα expression using a different sAPPα antibody and sAPPβ expression using 22C11 antibody.

Additional file 6. Figure S6. Enhanced APP phosphorylation decreases APP SUMOylation. EGFP-APPWT or EGFP-APPT668D phospho-mimicking mutant plasmid was co-transfected with HA-Ubc9 and Myc-SUMO1 or Myc-SUMO1∆GG plasmid to HEK293T cells. Forty-eight hours later, the cell lysates were immunoprecipitated with anti-EGFP antibody and immunoblotted with anti-SUMO1 antibody for determination of the APP SUMOylation level. Immunoblotting with antibodies against various tags was carried out to verify the transfection and expression of various plasmids. Experiments are in three repeats. The quantified result of APP SUMOylation. Data are mean±SEM. # *P *< 0.001.

Additional file 7. Figure S7. Melatonin decreases APP Thr-668 phosphorylation in APP/PS1 mice. EtOH was injected to WT or APP/PS1 mice, and melatonin was injected to APP/PS1 mice at one injection per day for 21 days consecutively. Animals were sacrificed 3 days after the last injection and their hippocampal tissue was subjected to western blot determination of p-Thr668 APP phosphorylation, total APP protein expression and transgenic human APP695 protein expression. The quantified result of APP Thr-668 phosphorylation level over that of total APP protein level. Data are mean±SEM. * *P *< 0.05.

Additional file 8. Figure S8. PSEN1 is SUMOylated in mouse hippocampus. Wild-type mice received intra-hippocampal EGFP-vector transfection or EGFP-PSEN1 transfection. The SUMO1 protein or the SUMO1∆GG mutant protein was added to the reaction to two groups of mice that received EGFP-PSEN1 transfection, respectively. Animals were sacrificed 48 h after plasmid transfection and their hippocampal tissue was subjected to the SUMOylation assay. Cell lysate was immunoprecipitated with anti-EGFP antibody and immunoblotted with anti-SUMO1 antibody. The SUMO-PSEN1 band is shown. The same lysate was also immunoprecipitated and immunoblotted with anti-EGFP antibody to confirm the transfection and expression of the plasmid. Experiments are in two repeats.

Additional file 9. Figure S9. APP SUMOylation regulates APP-CTFβ, APP-CTFα and sAPPβ expression, and the expression of sAPPβ is consistent with that of APP-CTFβ. Lenti-EGFP vector or Lenti-EGFP-SUMO1 or Lenti-EGFP-SUMO1∆GG plasmid was transducted to the hippocampus of WT or APP/PS1 mice as that described in Supplementary Figure 2. Their CA1 tissue was subjected to western blot analysis and APP-CTFbeta and APP-CTFalpha were detected using anti-APP C-terminal antibody. The membrane was stripped and re-probed with the sAPPβ antibody. Experiments are in two repeats.

Additional file 10.

## Data Availability

No datasets were generated or analysed during the current study.

## Abbreviations

APP: Amyloid precursor protein
AD: Alzheimer's disease
sAPPα: Soluble amyloid precursor protein alpha
sAPPβ: soluble amyloid precursor protein beta
AICD: Amyloid precursor protein intracellular domain
Aβ: Amyloid beta
BACE1: Beta-secretase 1
SUMO: Small ubiquitin-like modifier
PS1: Presenilin-1
PBS: Phosphate buffered saline
EGFP: Enhanced green fluorescent protein
siRNA: Small interference RNA
i.p.: Intraperitoneal injection
IP: Immunoprecipitation
SDS: Sodium dodecyl sulfate polyacrylamide
PVDF: Polyvinylidene difluoride
BSA: Bovine serum albumin
