# Supplementary figures and images for: APP SUMOylation prevents BACE1 cleavage of APP and increases BACE1 degradation to promote the nonamyloidogenic pathway
Source: Mol Med. 2025 Sep 29;31:301. doi: 10.1186/s10020-025-01354-8 (PMC12482303; doi:10.1186/s10020-025-01354-8)

## Supplementary Figure 1

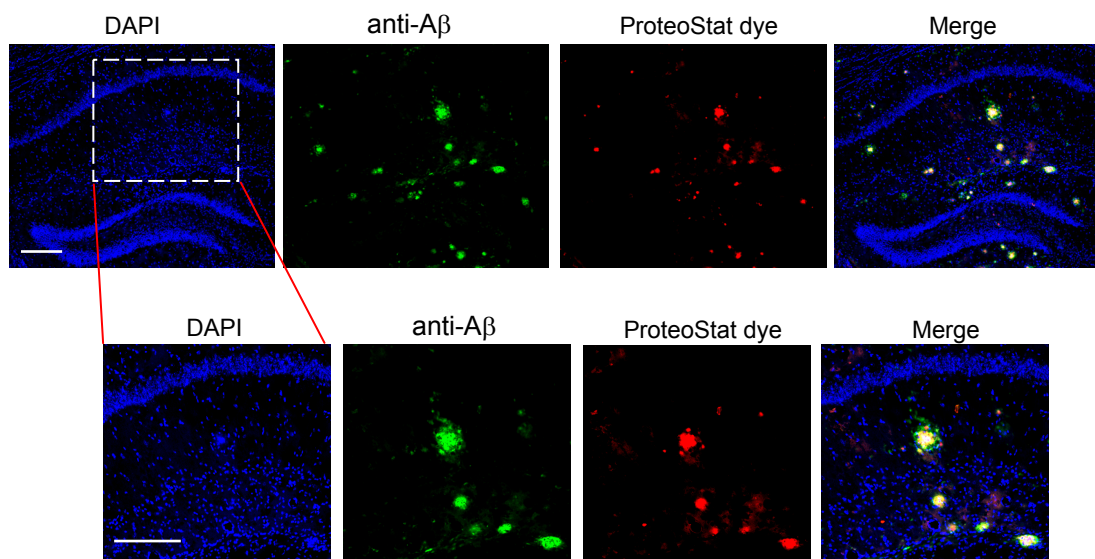

Supplement: Supplementary file 1 — Additional file 1. Figure S1. ProteoStat dye and Aβ staining co-localize in the hippocampus. The mouse hippocampal tissue slice was subjected to immunohistochemistry staining of ProteoStat dye and Aβ. The images of ProteoStat dye staining and Aβ staining and their merged image are shown. Scale bar equals 200 μm for both the upper panel and lower panel. [file 10020_2025_1354_MOESM1_ESM.pdf]

## Supplementary Figure 2

**A**

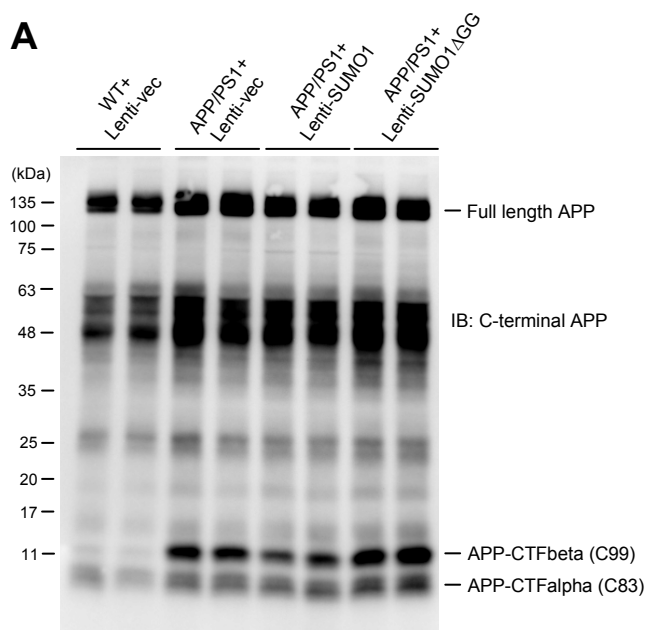

**B**

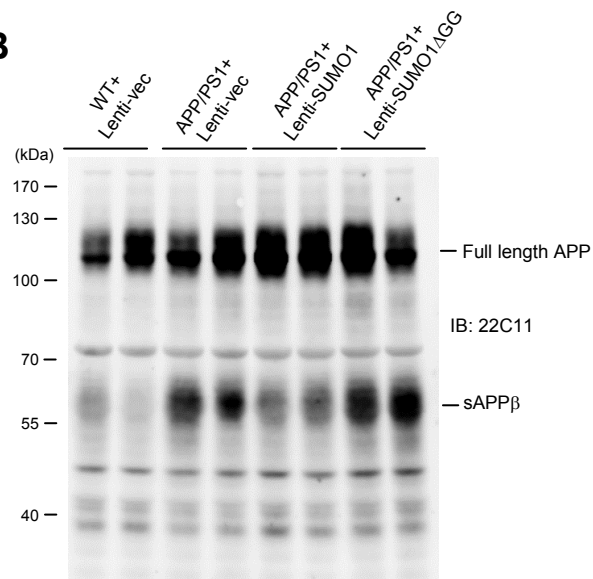

**C**

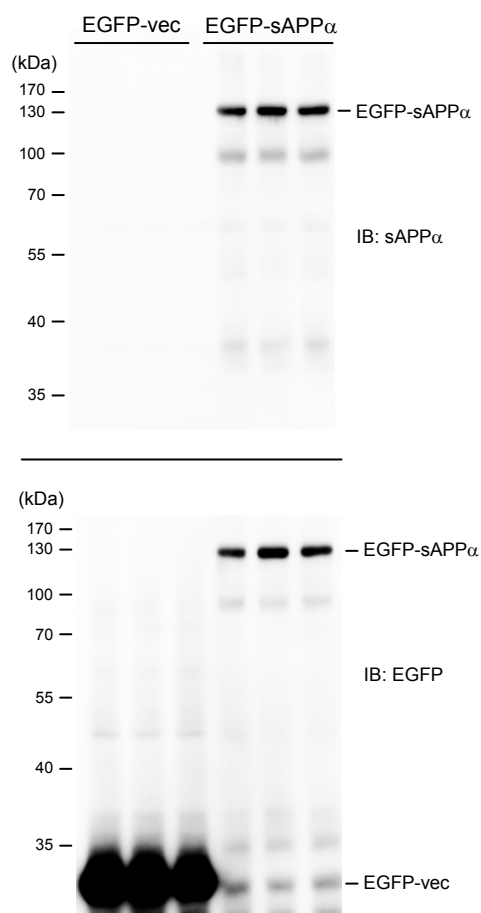

**D**

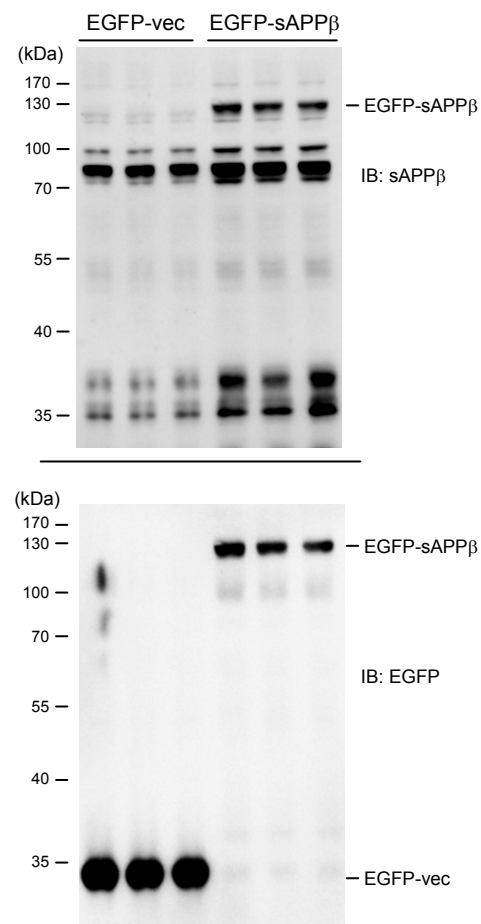

Supplement: Supplementary file 2 — Additional file 2. Figure S2. APP SUMOylation regulates APP-CTFalpha, APP-CTFbeta and sAPPβ expression. Lenti-EGFP vector or Lenti-EGFP-SUMO1 or Lenti-EGFP-SUMO1∆GG plasmid was transducted to the hippocampus of WT or APP/PS1 mice. Their CA1 tissue was dissected out and the tissue lysate was subjected to western blot determination of APP-CTFalpha and APP-CTFbeta expression using anti-APP C-terminal antibody. The same tissue lysate was also subjected to western blot determination of sAPP expression using 22C11 antibody. HEK293T cells were transfected with EGFP-vector or EGFP-sAPPα plasmid. Cell lysates were subjected to immunoblotting with anti-sAPPα antibody to determine the expression of EGFP-sAPPα. Immunoblotting with anti-EGFP antibody was used to verify plasmid transfection and expression. HEK293T cells were transfected with EGFP-vector or EGFP-sAPP plasmid. Cell lysates were subjected to immunoblotting with anti-sAPP antibody to determine the expression of EGFP-sAPP. Immunoblotting with anti-EGFP antibody was used to verify plasmid transfection and expression. [file 10020_2025_1354_MOESM2_ESM.pdf]

# Supplementary Figure 3

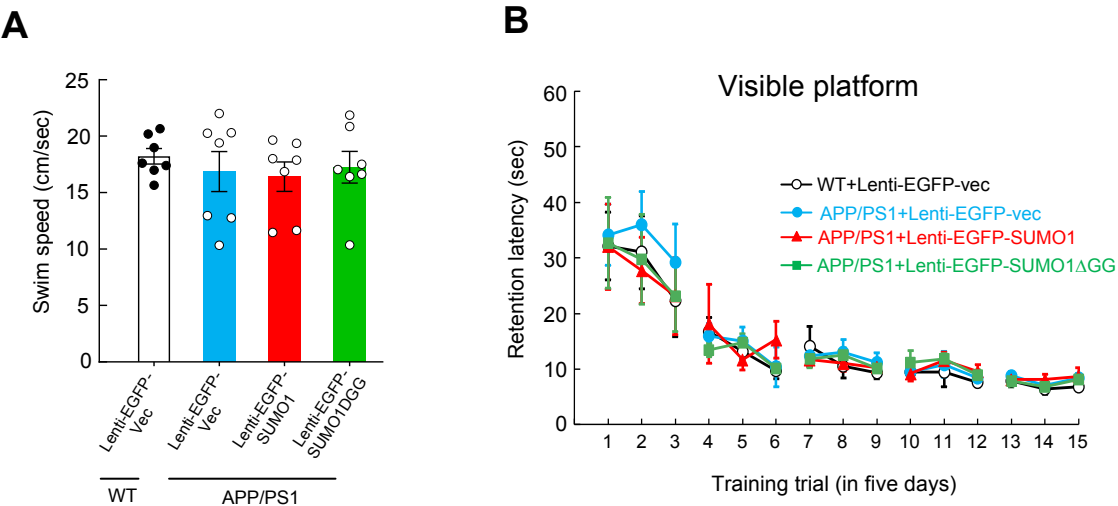

Supplement: Supplementary file 3 — Additional file 3. Figure S3. Lenti-EGFP vector, Lenti-EGFP-SUMO1 or Lenti-EGFP-SUMO1ΔGG transduction do not affect swim speed of probe trial test in APP/PS1 mice. Animals received intra-hippocampal transduction of Lenti-EGFP vector, Lenti-EGFP-SUMO1 vector or Lenti-EGFP-SUMO1ΔGG vector and their swim speeds for the probe trial test are shown. Visible platform performance from the same animals is shown. Data are mean±SEM. [file 10020_2025_1354_MOESM3_ESM.pdf]

## Supplementary Figure 4

**A**

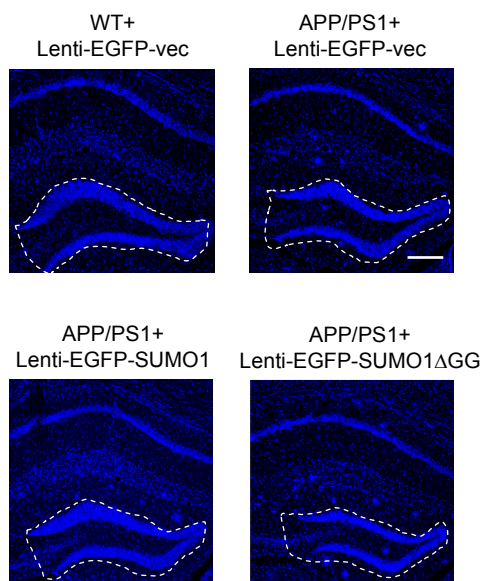

**B**

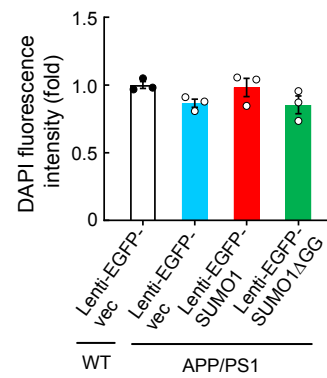

Supplement: Supplementary file 4 — Additional file 4. Figure S4. DAPI staining intensity is not altered in APP/PS1 mice receiving. Lenti-EGFP-vector, Lenti-EGFP-SUMO1 or Lenti-EGFP-SUMO1∆GG transduction to the hippocampus. WT mice received Lenti-EGFP-vector transduction and APP/PS1 mice received Lenti-EGFP-vector, Lenti-EGFP-SUMO1 or Lenti-EGFP-SUMO1∆GG transduction as described in Figure 5E. Immunohistochemistry of DAPI staining was performed and the fluorescence intensity of DAPI staining in the marked area was measured. Scale bar equals 200 μm. The quantified result of DAPI fluorescence intensity for each group. Data are expressed as mean±SEM. [file 10020_2025_1354_MOESM4_ESM.pdf]

## Supplementary Figure 5

**A**

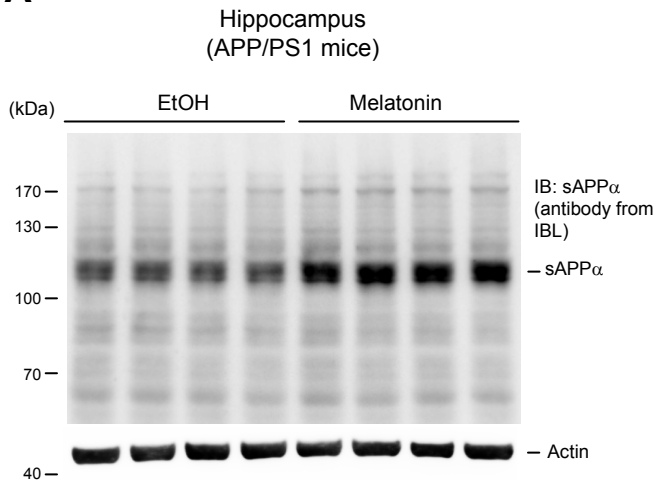

**B**

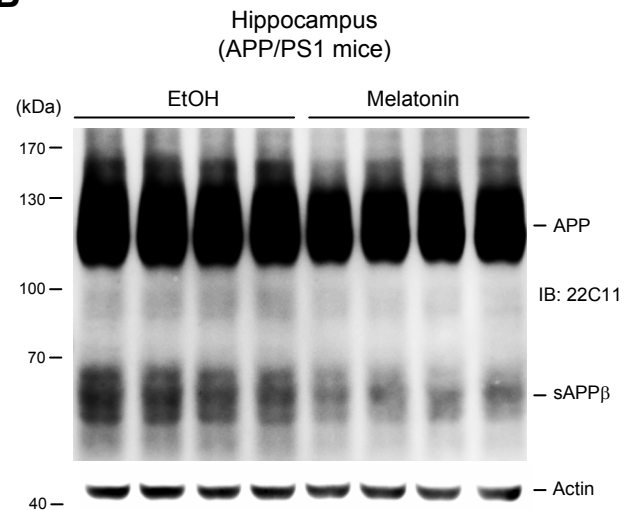

**C**

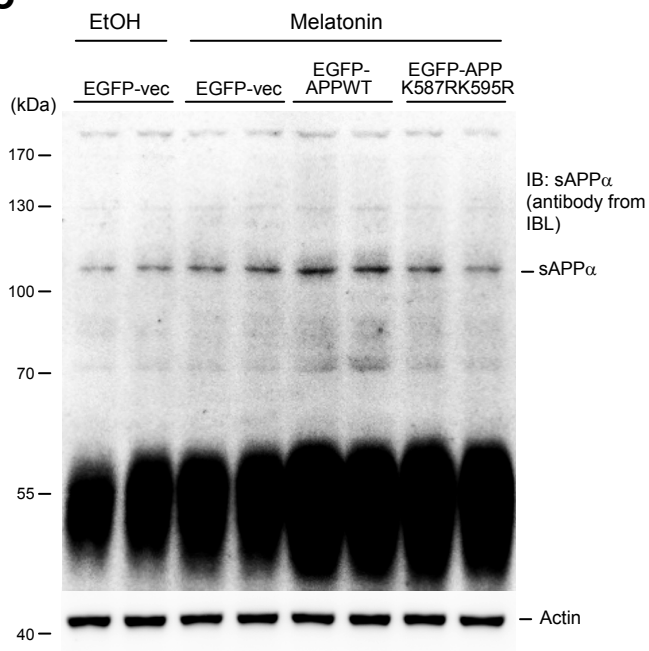

**D**

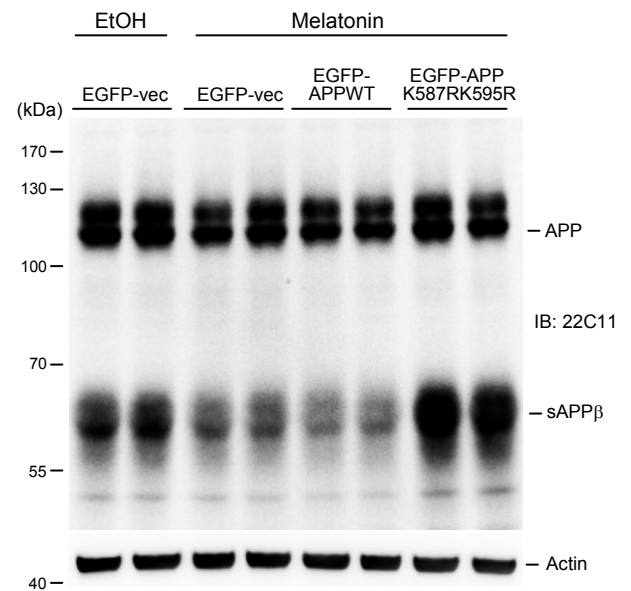

Supplement: Supplementary file 5 — Additional file 5. Figure S5. Melatonin increases sAPPα expression and decreases sAPPβ expression and these effects were blocked by APP sumo-mutant transfection Mice were divided to two groups and received intraperitoneal EtOH or melatonin injection for 21 days consecutively. They were sacrificed 3 days after the last injection and their hippocampal tissue was subjected to western blot determinations of sAPPα expression using a different sAPPα antibody and sAPPβ expression using 22C11 antibody. Wild-type mice were divided to four groups and received intra-hippocampal EGFP-vector+EtOH, EGFP-vector+melatonin, EGFP-APPWT+melatonin or EGFP-APPK587RK595R+melatonin plasmid transfection and injection. EtOH or melatonin was administered 47 h after plasmid transfection and animals were sacrificed 1 h after EtOH/melatonin injection. Their hippocampal tissue was subjected to western blot determination of sAPPα expression using a different sAPPα antibody and sAPPβ expression using 22C11 antibody. [file 10020_2025_1354_MOESM5_ESM.pdf]

# Supplementary Figure 6

**A**

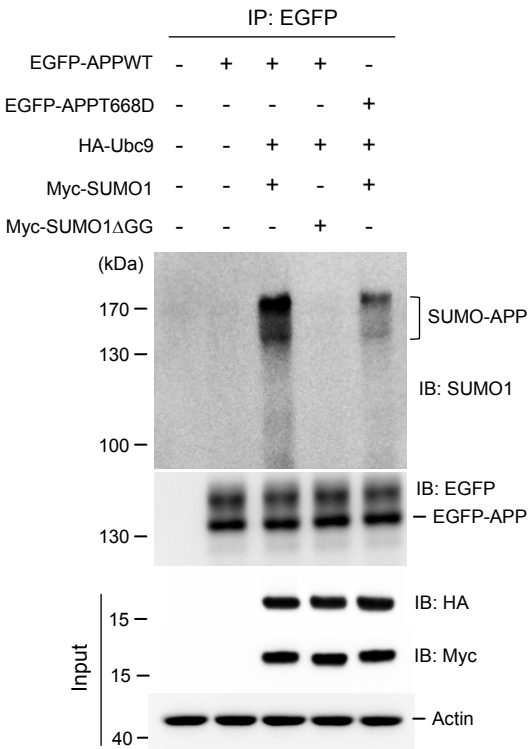

**B**

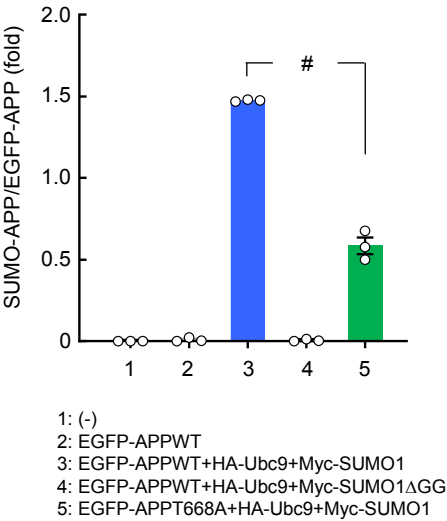

Supplement: Supplementary file 6 — Additional file 6. Figure S6. Enhanced APP phosphorylation decreases APP SUMOylation. EGFP-APPWT or EGFP-APPT668D phospho-mimicking mutant plasmid was co-transfected with HA-Ubc9 and Myc-SUMO1 or Myc-SUMO1∆GG plasmid to HEK293T cells. Forty-eight hours later, the cell lysates were immunoprecipitated with anti-EGFP antibody and immunoblotted with anti-SUMO1 antibody for determination of the APP SUMOylation level. Immunoblotting with antibodies against various tags was carried out to verify the transfection and expression of various plasmids. Experiments are in three repeats. The quantified result of APP SUMOylation. Data are mean±SEM. # P < 0.001. [file 10020_2025_1354_MOESM6_ESM.pdf]

# Supplementary Figure 7

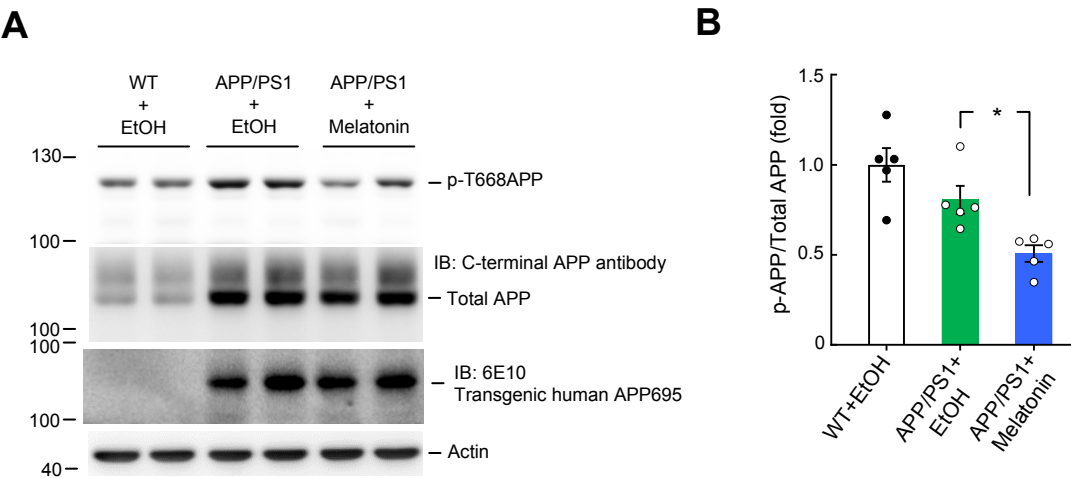

Supplement: Supplementary file 7 — Additional file 7. Figure S7. Melatonin decreases APP Thr-668 phosphorylation in APP/PS1 mice. EtOH was injected to WT or APP/PS1 mice, and melatonin was injected to APP/PS1 mice at one injection per day for 21 days consecutively. Animals were sacrificed 3 days after the last injection and their hippocampal tissue was subjected to western blot determination of p-Thr668 APP phosphorylation, total APP protein expression and transgenic human APP695 protein expression. The quantified result of APP Thr-668 phosphorylation level over that of total APP protein level. Data are mean±SEM. * P < 0.05. [file 10020_2025_1354_MOESM7_ESM.pdf]

# Supplementary Figure 8

SUMOylation assay in WT mice

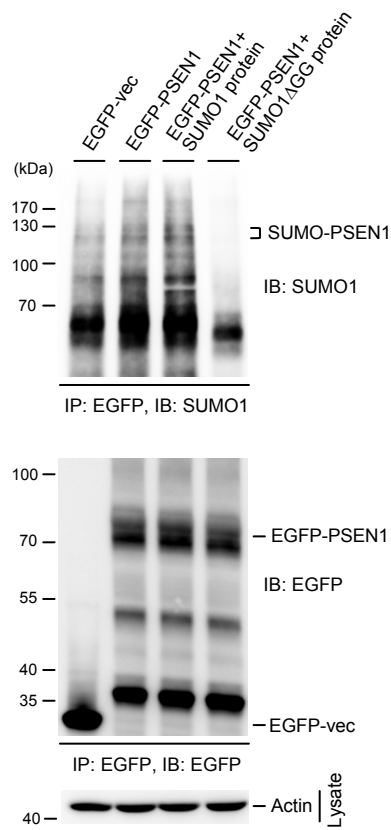

Supplement: Supplementary file 8 — Additional file 8. Figure S8. PSEN1 is SUMOylated in mouse hippocampus. Wild-type mice received intra-hippocampal EGFP-vector transfection or EGFP-PSEN1 transfection. The SUMO1 protein or the SUMO1∆GG mutant protein was added to the reaction to two groups of mice that received EGFP-PSEN1 transfection, respectively. Animals were sacrificed 48 h after plasmid transfection and their hippocampal tissue was subjected to the SUMOylation assay. Cell lysate was immunoprecipitated with anti-EGFP antibody and immunoblotted with anti-SUMO1 antibody. The SUMO-PSEN1 band is shown. The same lysate was also immunoprecipitated and immunoblotted with anti-EGFP antibody to confirm the transfection and expression of the plasmid. Experiments are in two repeats. [file 10020_2025_1354_MOESM8_ESM.pdf]

# Supplementary Figure 9

Set #1

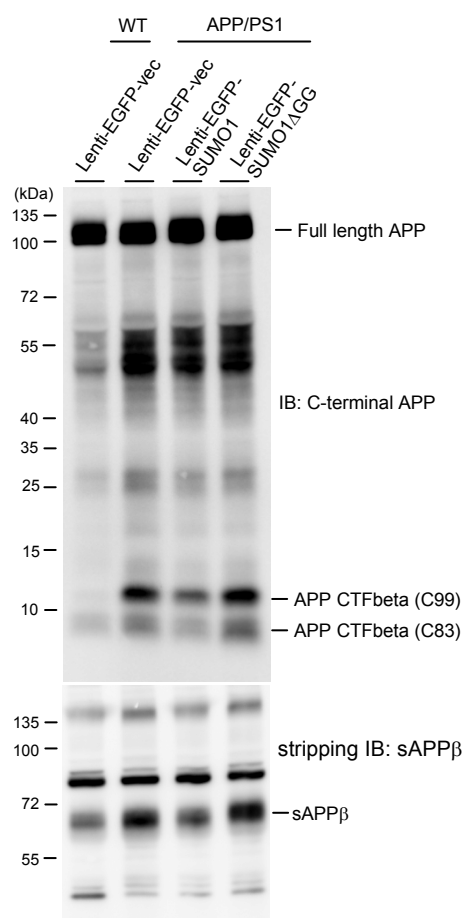

Set #2

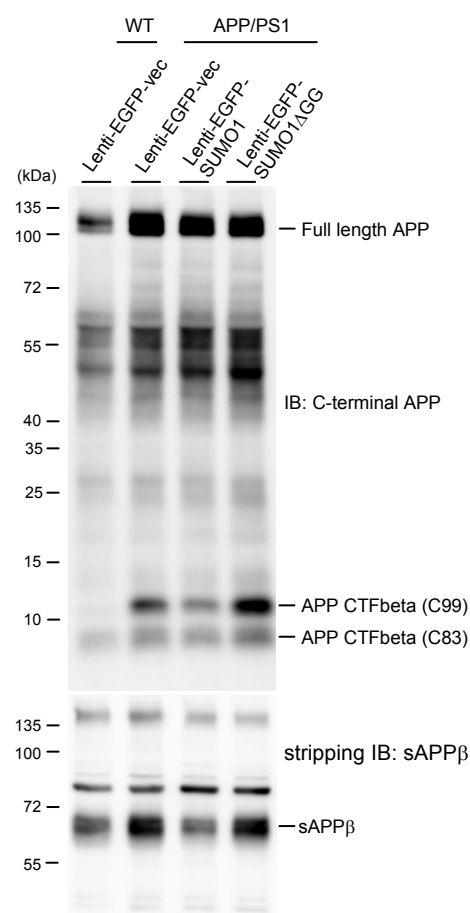

Supplement: Supplementary file 9 — Additional file 9. Figure S9. APP SUMOylation regulates APP-CTFβ, APP-CTFα and sAPPβ expression, and the expression of sAPPβ is consistent with that of APP-CTFβ. Lenti-EGFP vector or Lenti-EGFP-SUMO1 or Lenti-EGFP-SUMO1∆GG plasmid was transducted to the hippocampus of WT or APP/PS1 mice as that described in Supplementary Figure 2. Their CA1 tissue was subjected to western blot analysis and APP-CTFbeta and APP-CTFalpha were detected using anti-APP C-terminal antibody. The membrane was stripped and re-probed with the sAPPβ antibody. Experiments are in two repeats. [file 10020_2025_1354_MOESM9_ESM.pdf]
