# Supplementary material for: APP SUMOylation prevents BACE1 cleavage of APP and increases BACE1 degradation to promote the nonamyloidogenic pathway
Source: Mol Med. 2025 Sep 29;31:301. doi: 10.1186/s10020-025-01354-8 (PMC12482303; doi:10.1186/s10020-025-01354-8)

Supplementary Figure 10
Figure 1 original blot

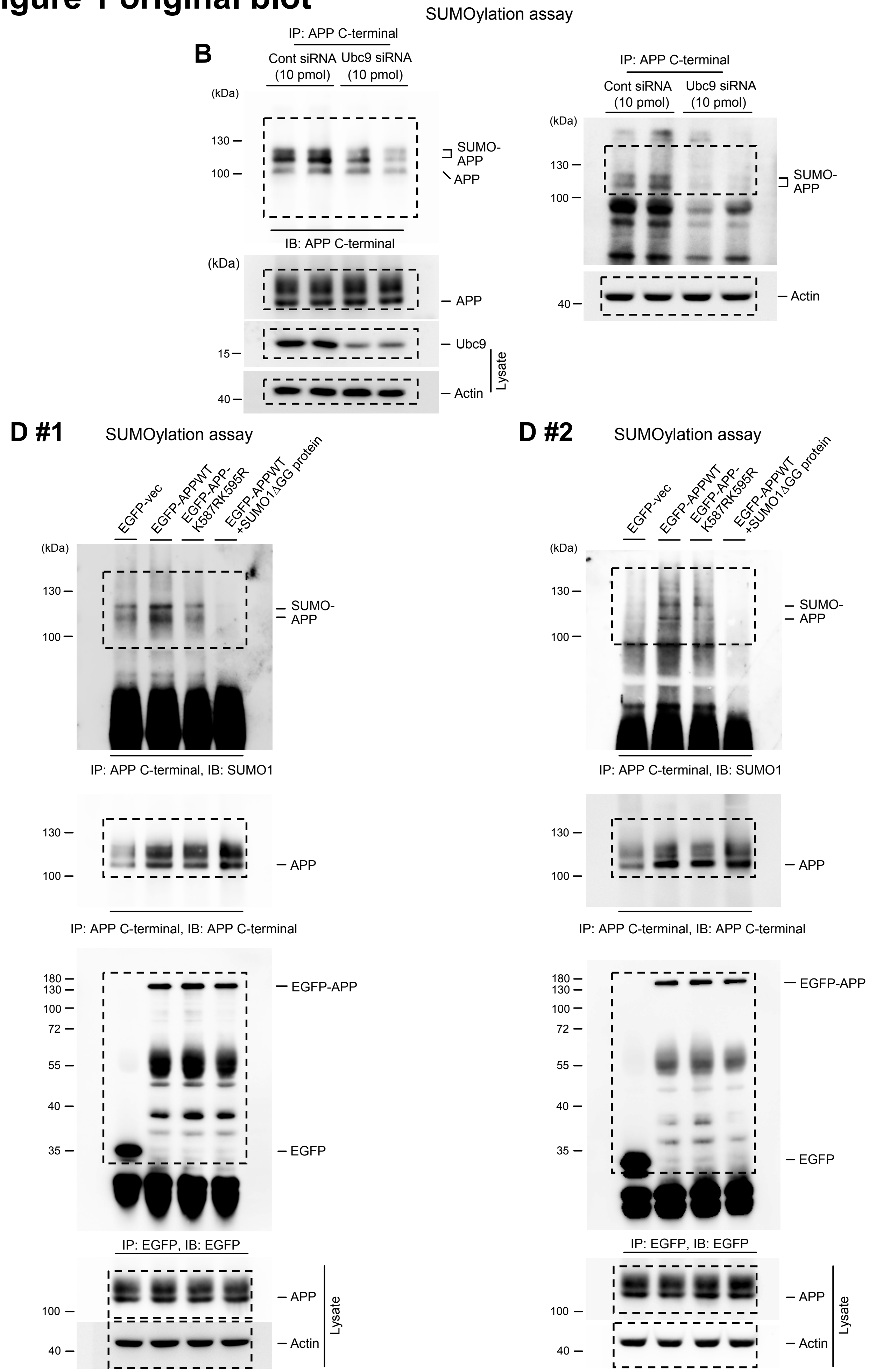

Figure 1 original blot (continue)

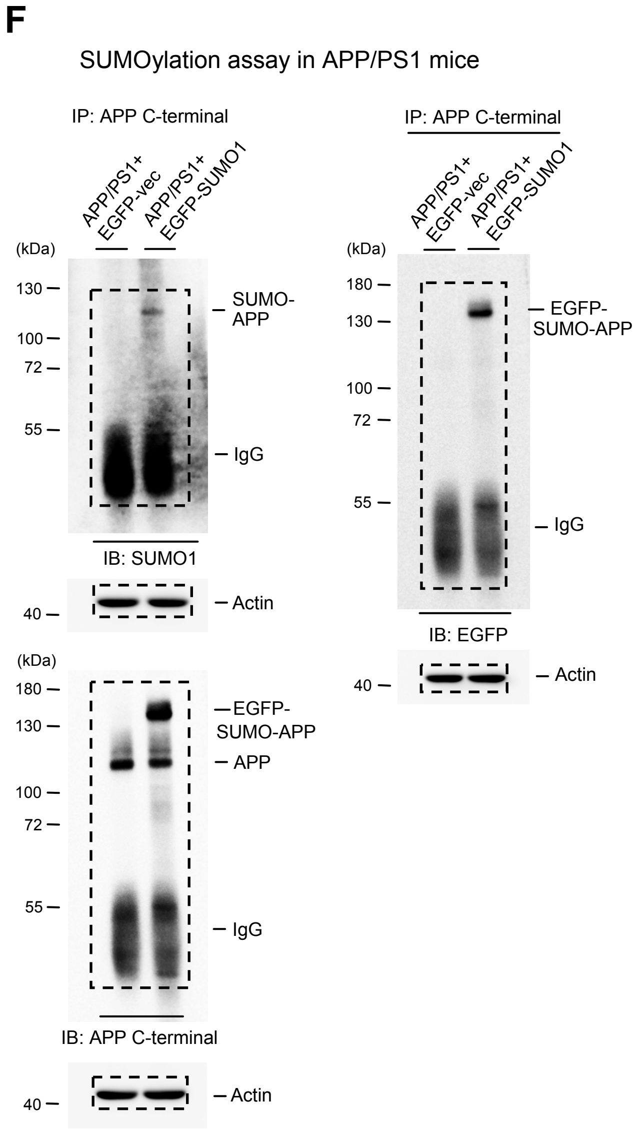

Figure 2 original blot

A #1

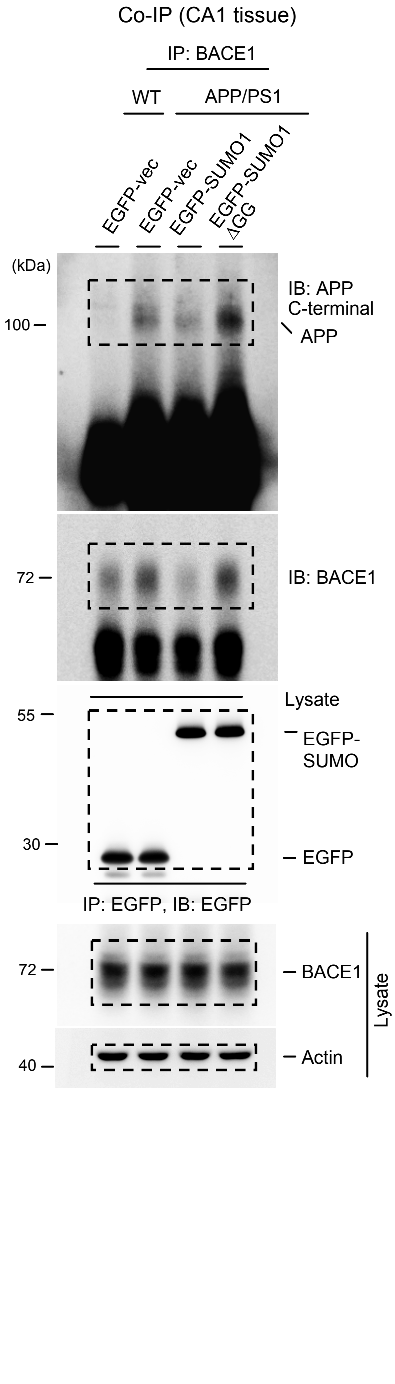

A #2

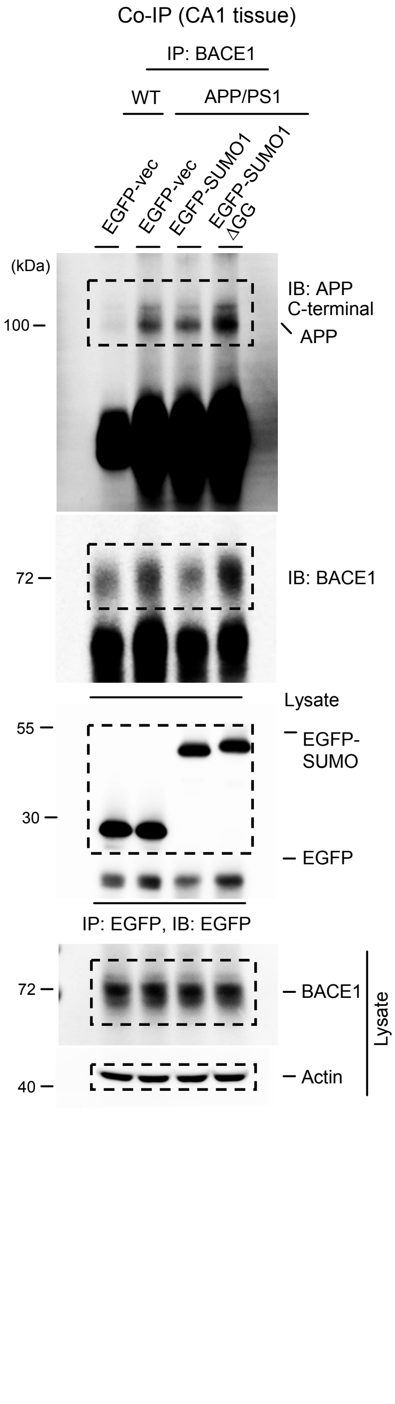

Figure 2 original blot (continue)

A #1

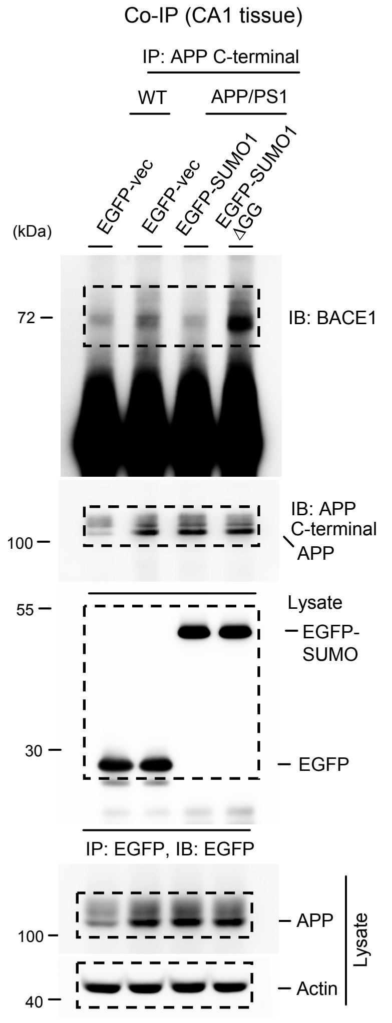

A #2

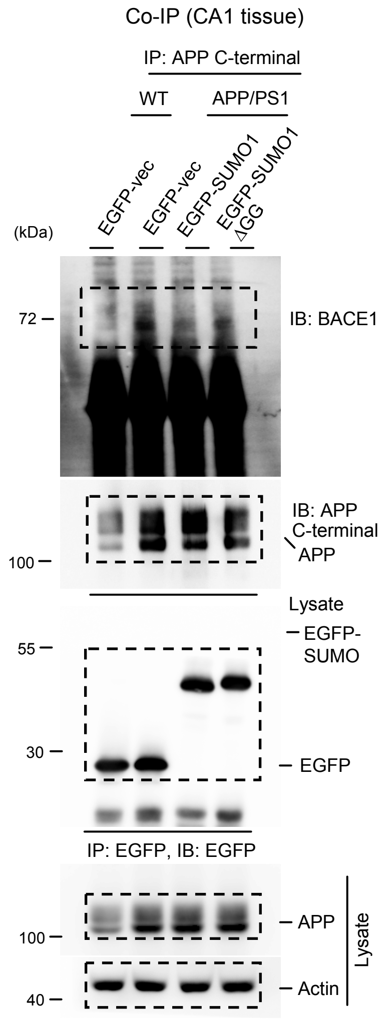

Figure 2 original blot (continue)

C #1

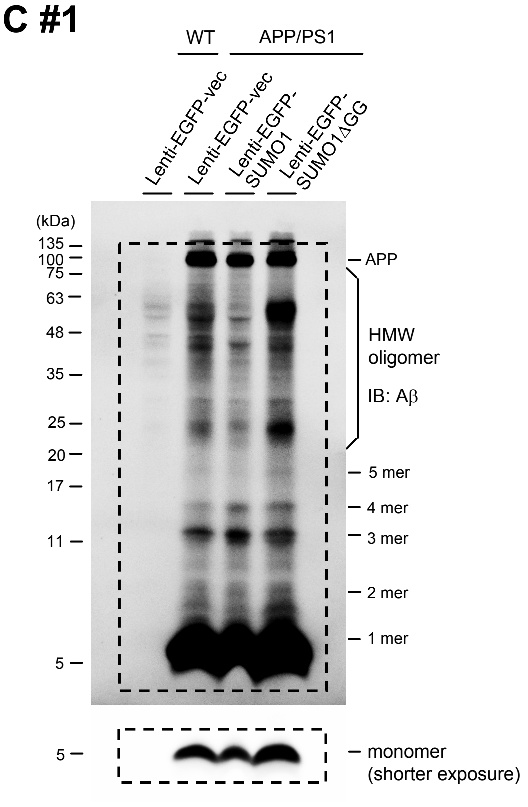

C #2

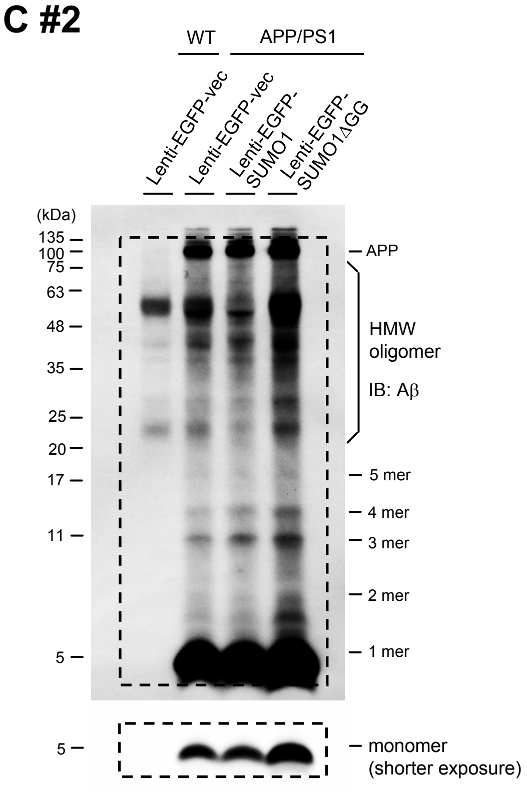

D #1

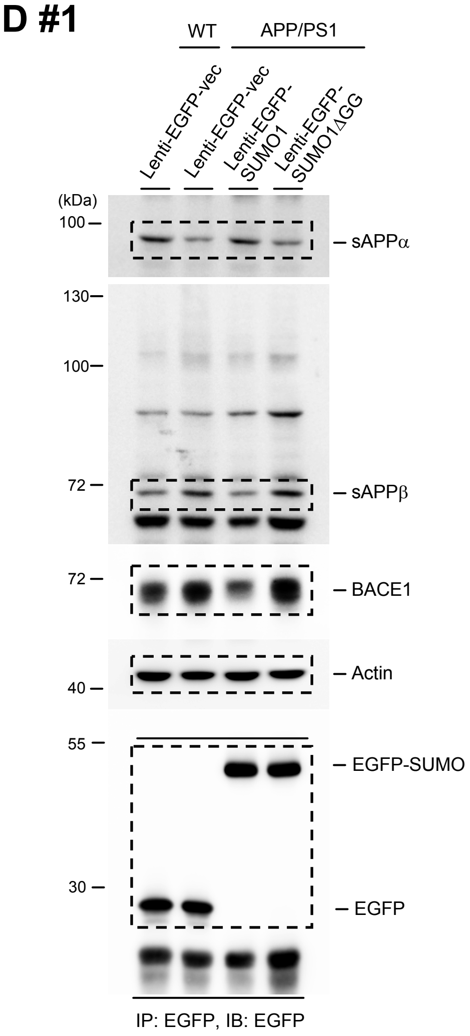

D #2

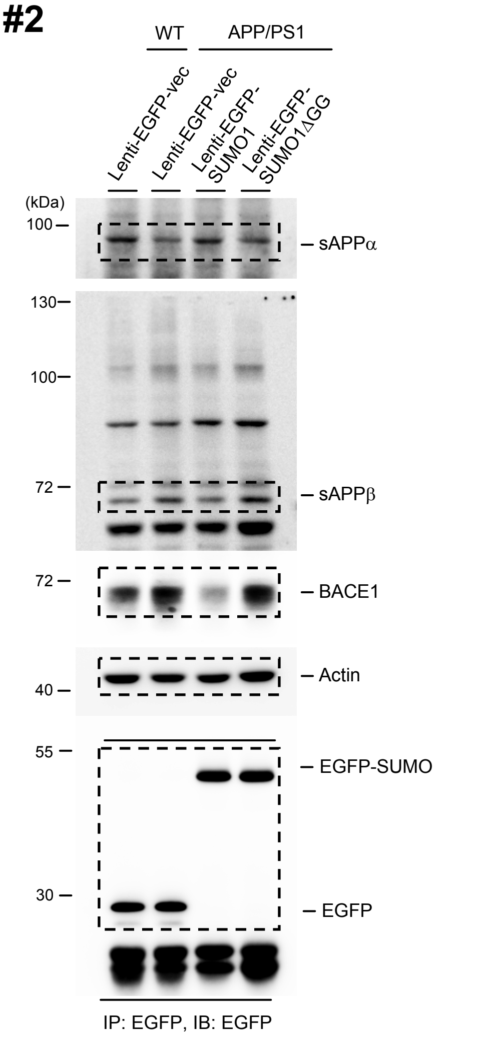

Figure 3 original blot

A #1

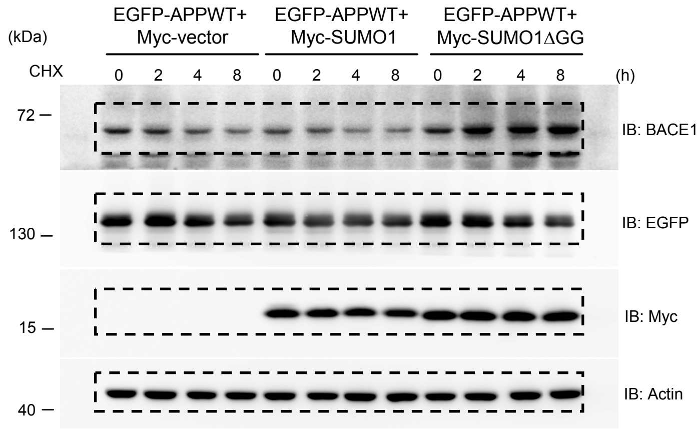

A #2

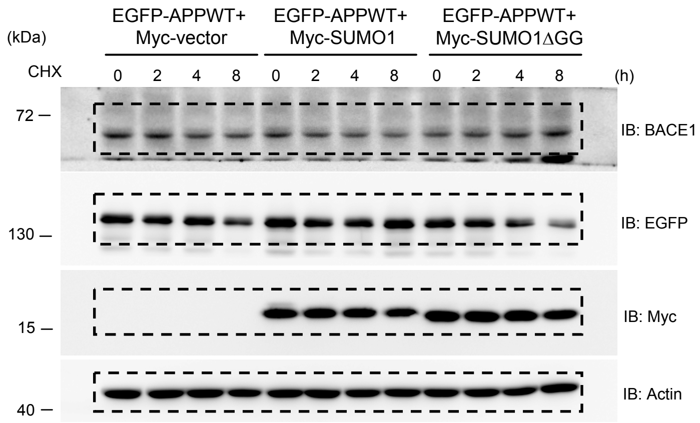

C

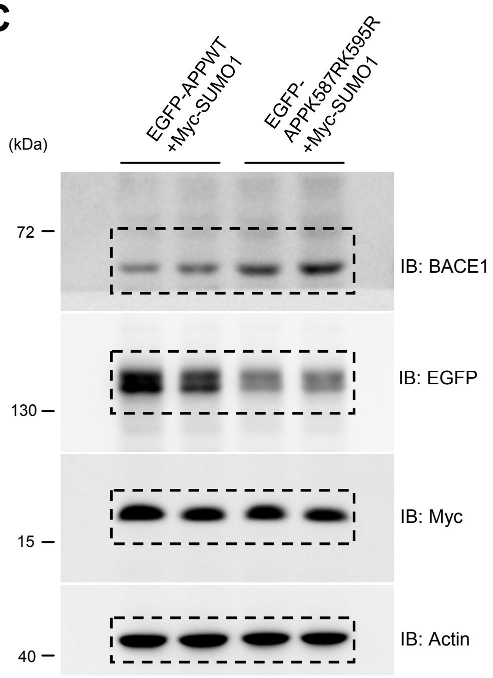

Figure 5 original blot

A #1

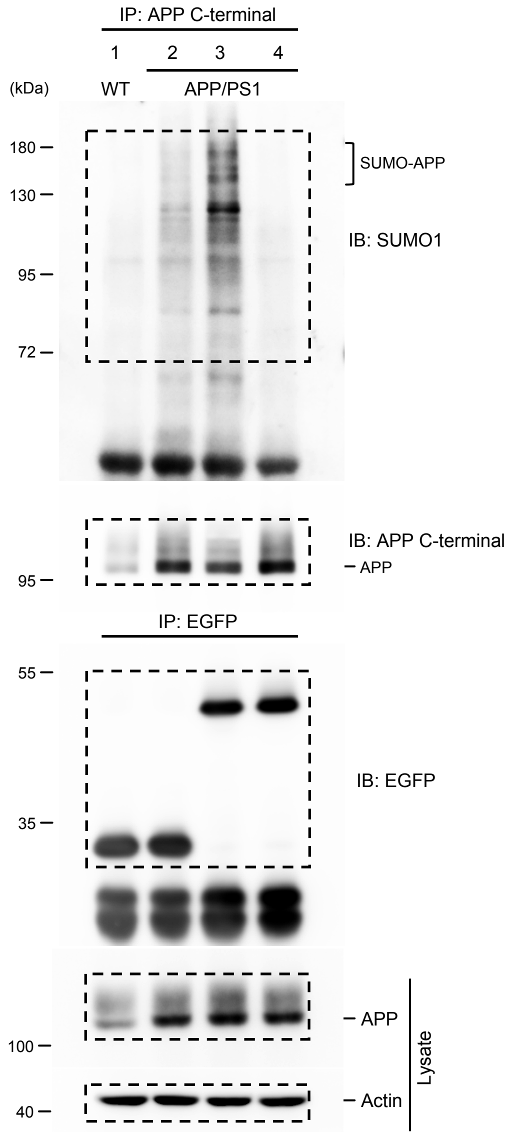

A #2

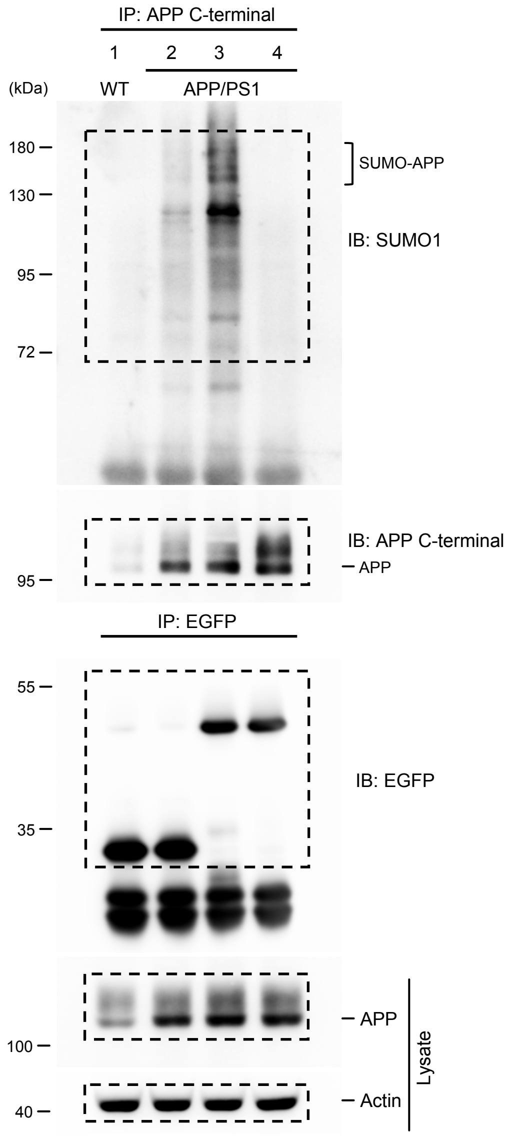

Figure 5 original blot (continue)

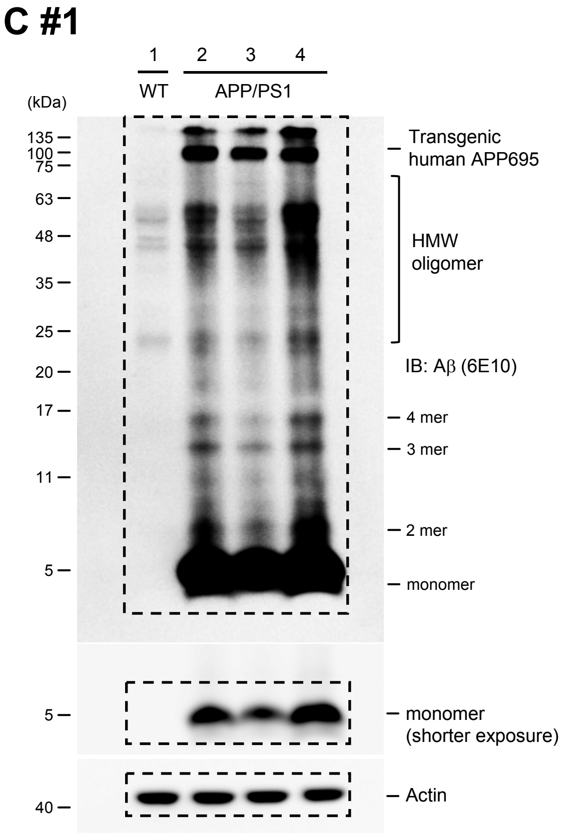

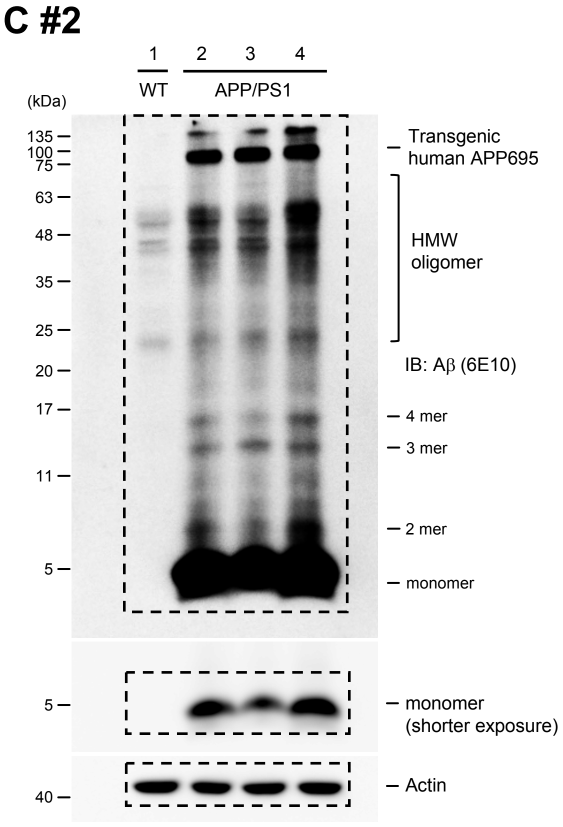

Figure 6 original blot

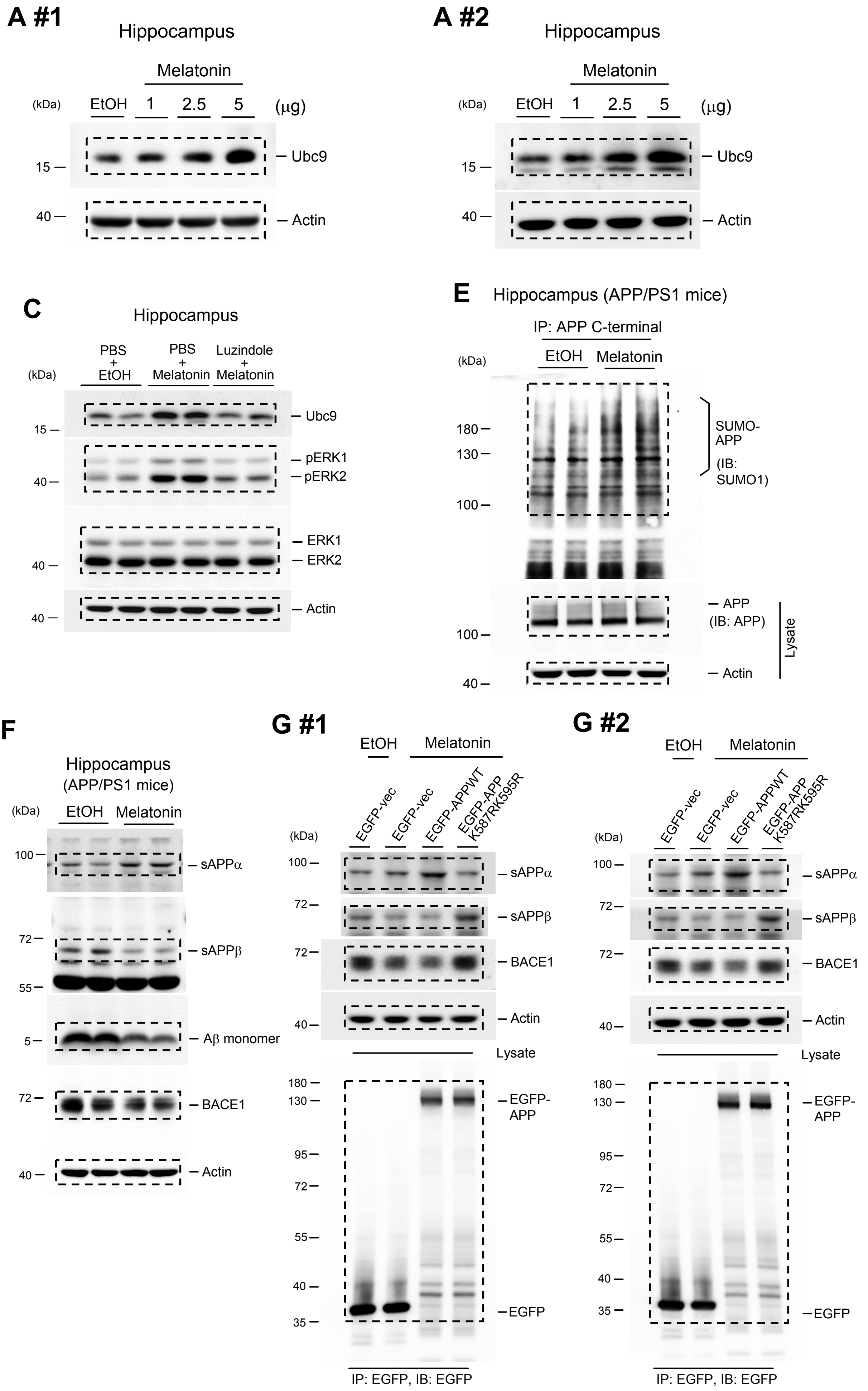

Figure 7 original blot

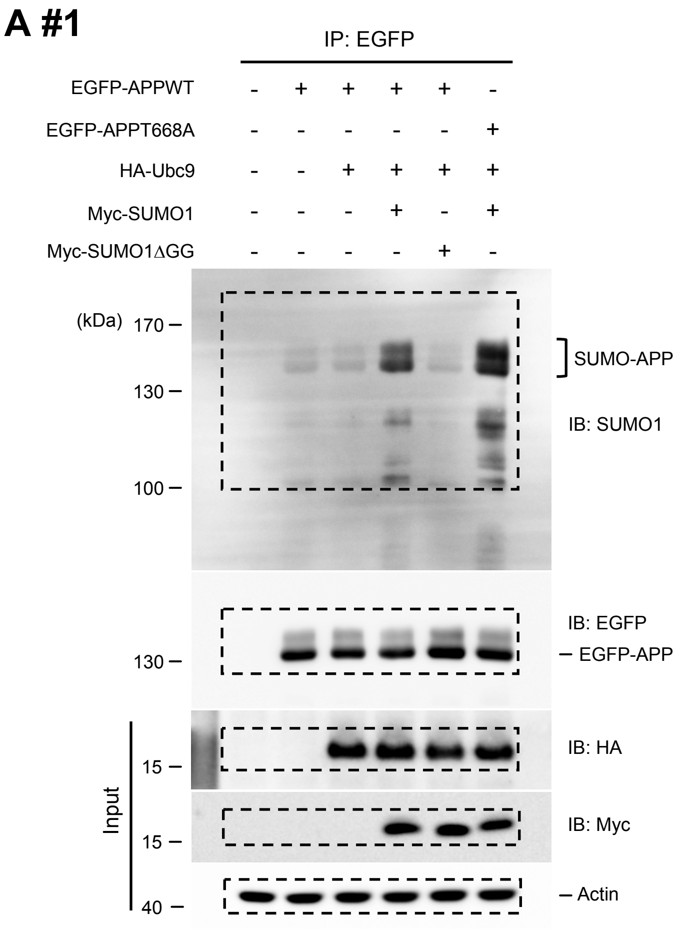

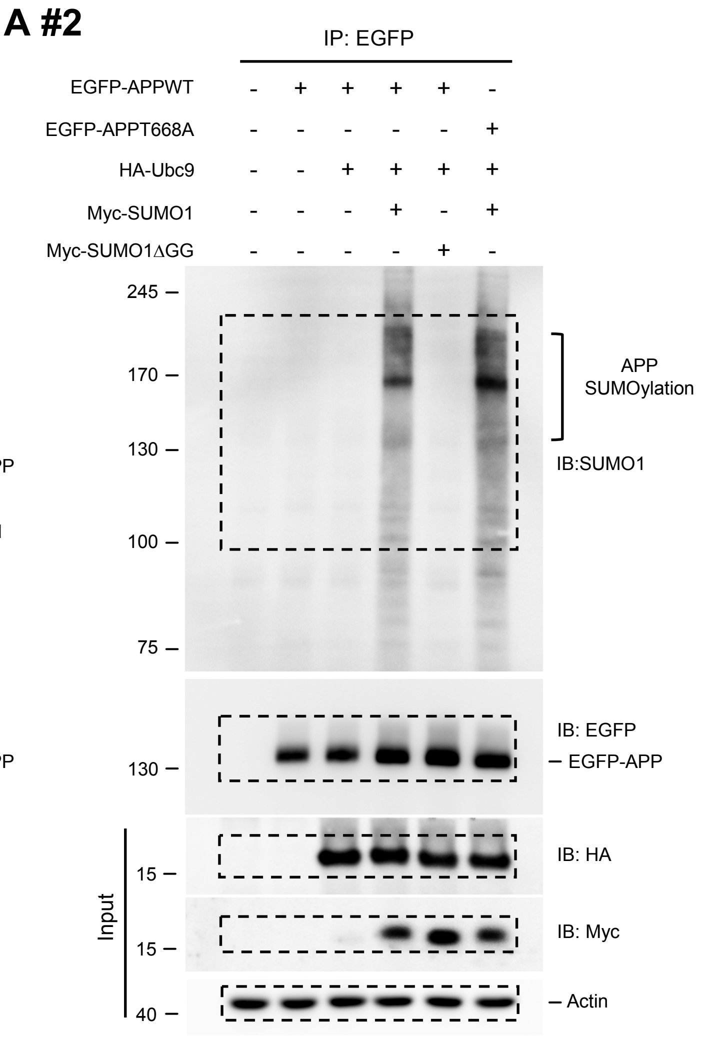

Figure 7 original blot (continue)

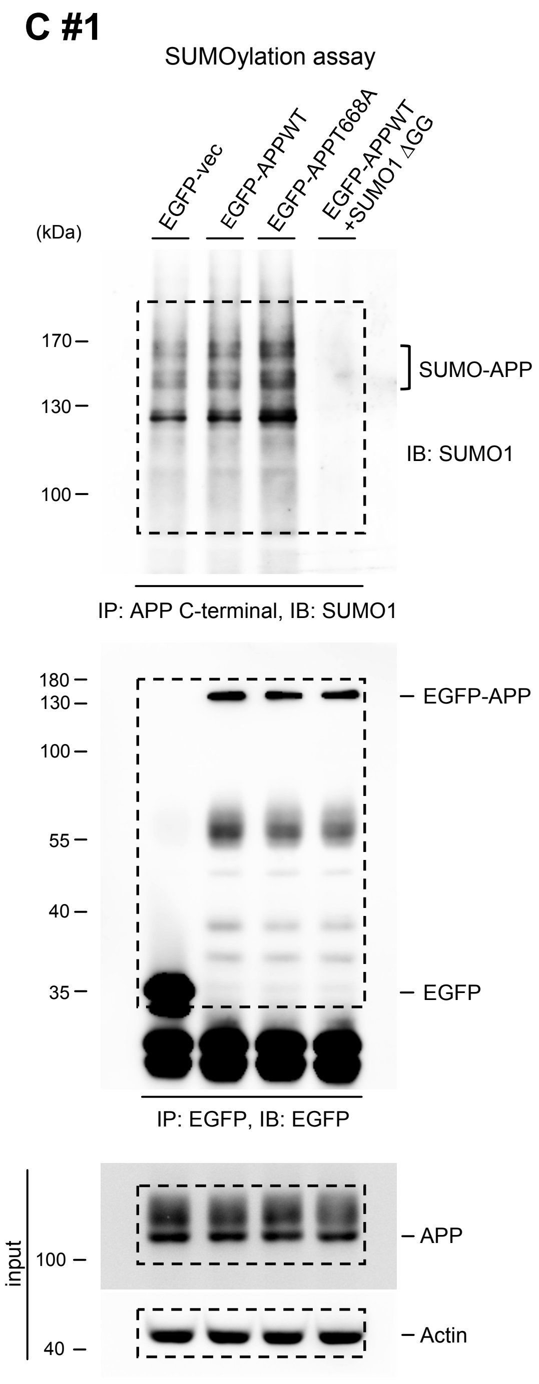

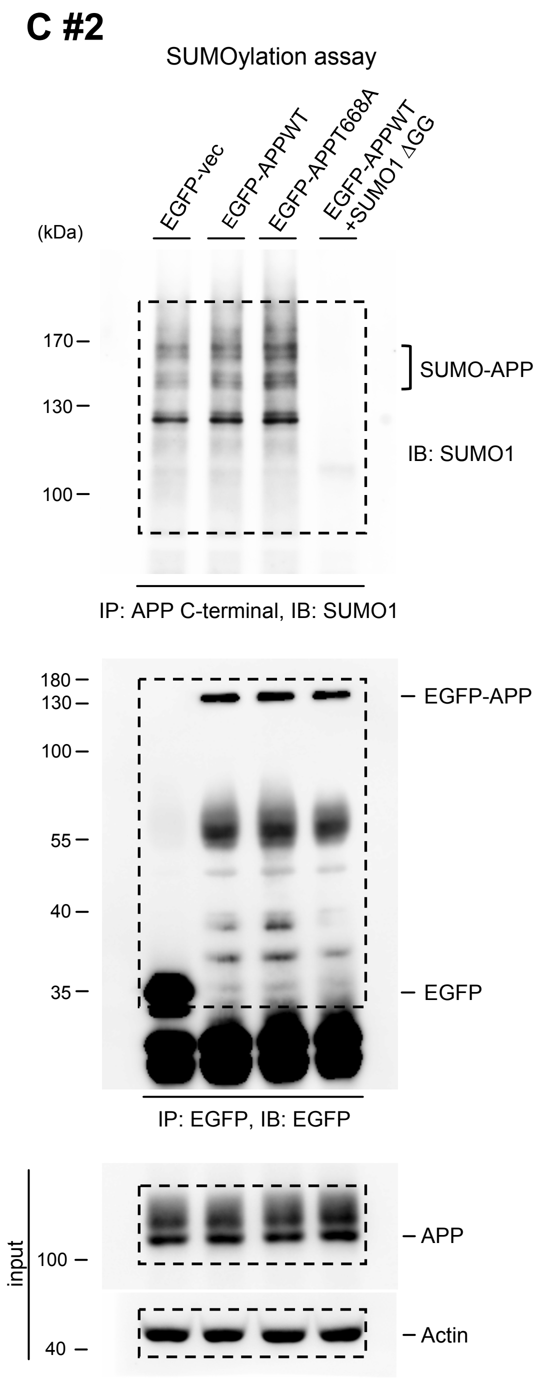

Figure 7 original blot (continue)

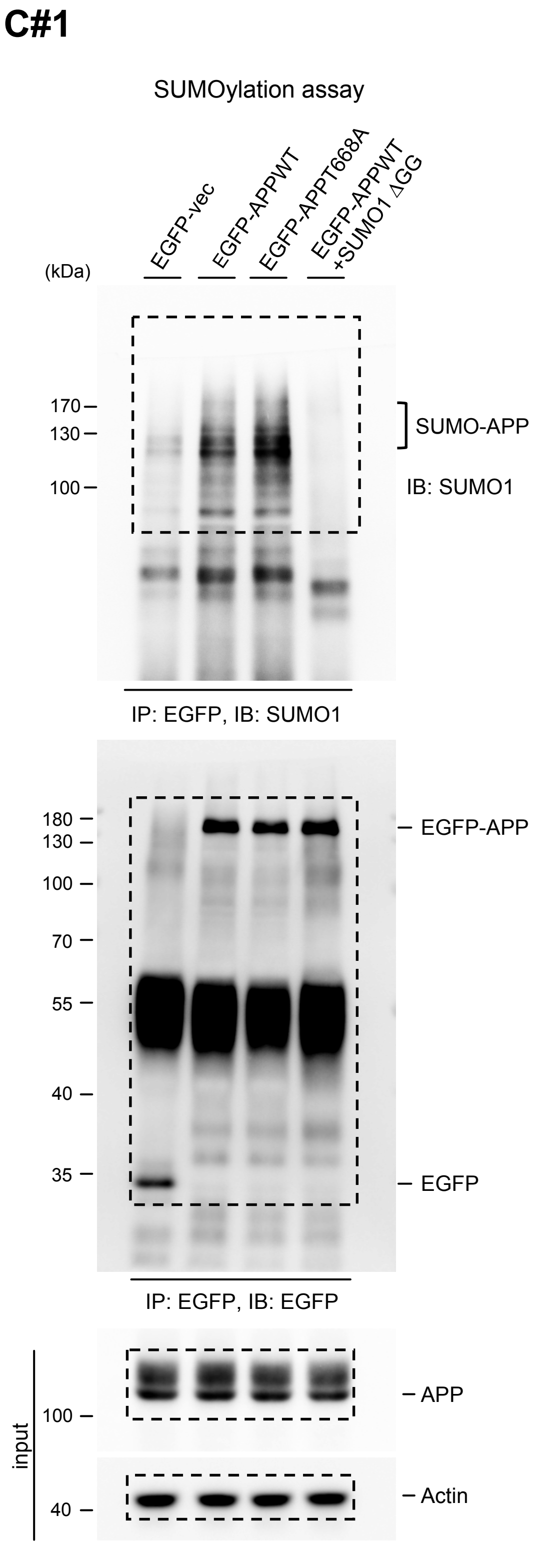

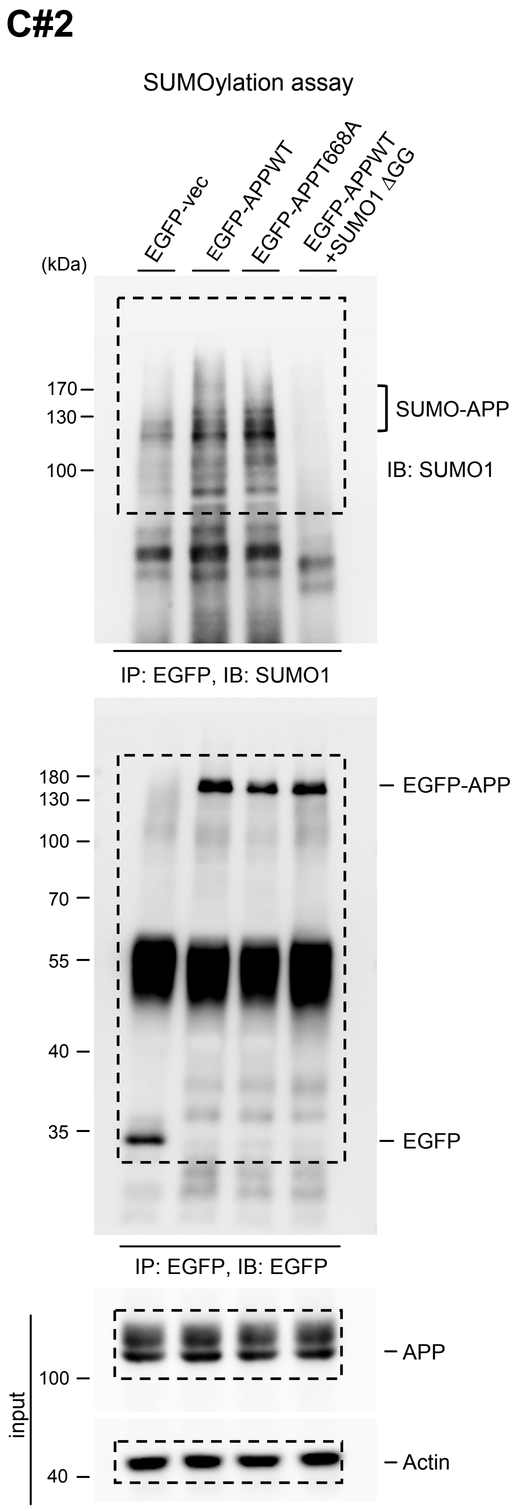

Supplementary Figure 2 original blot

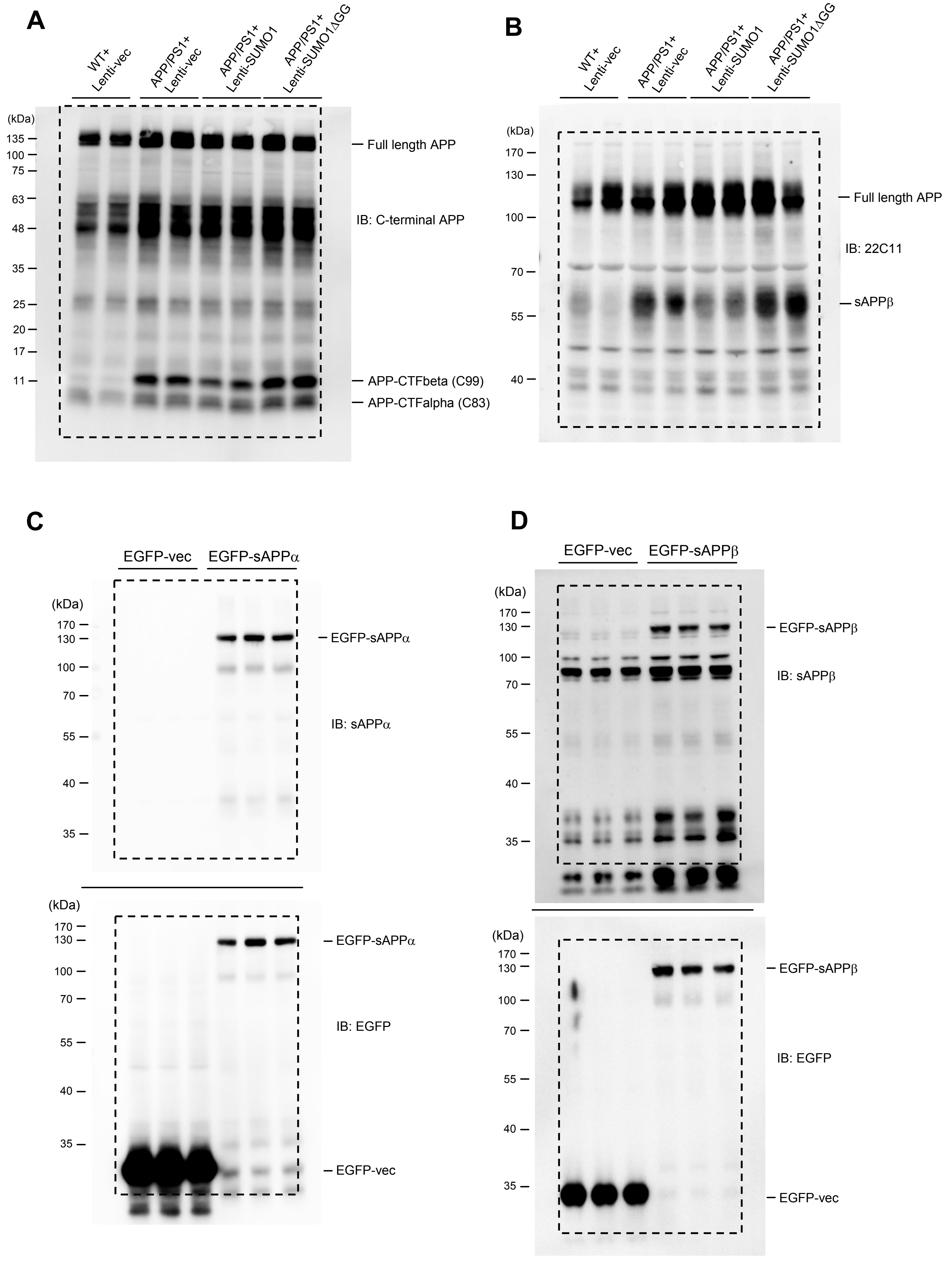

Supplementary Figure 5 original blot

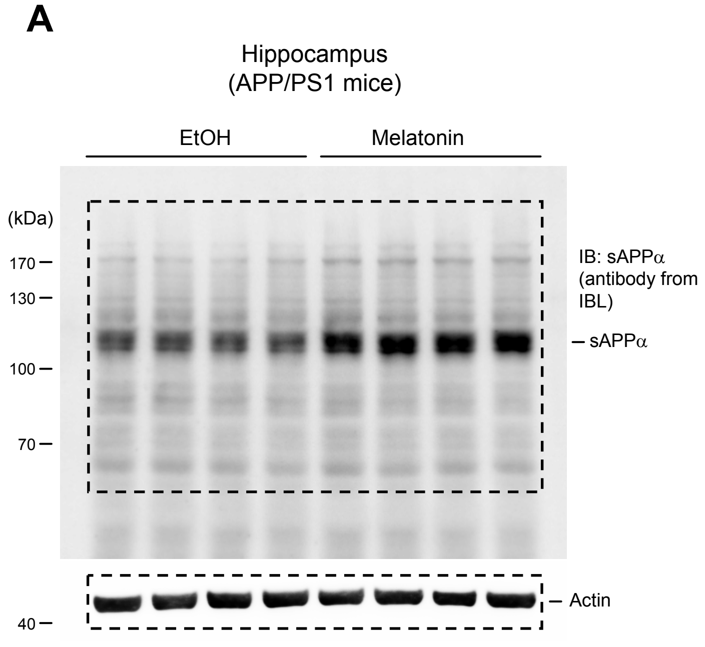

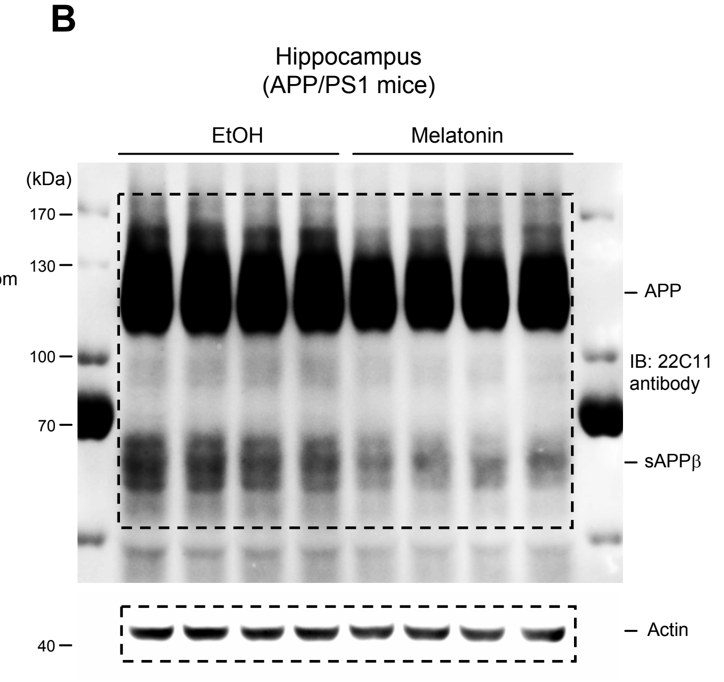

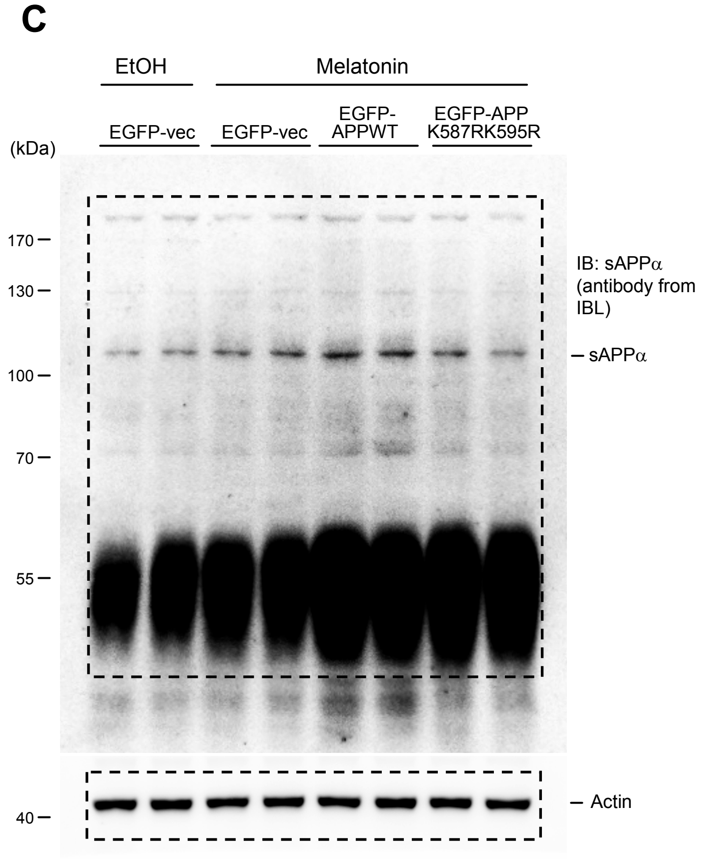

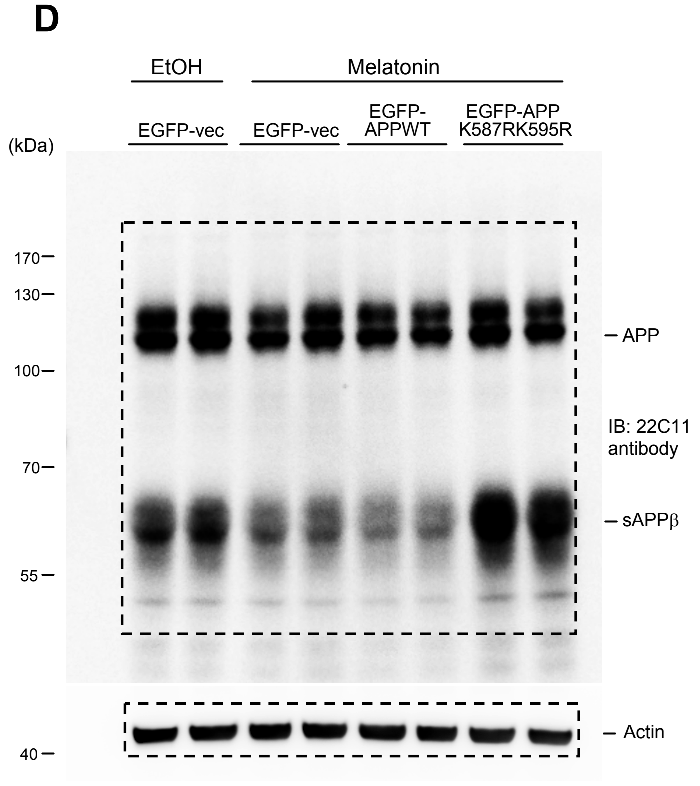

Supplementary Figure 6 original blot

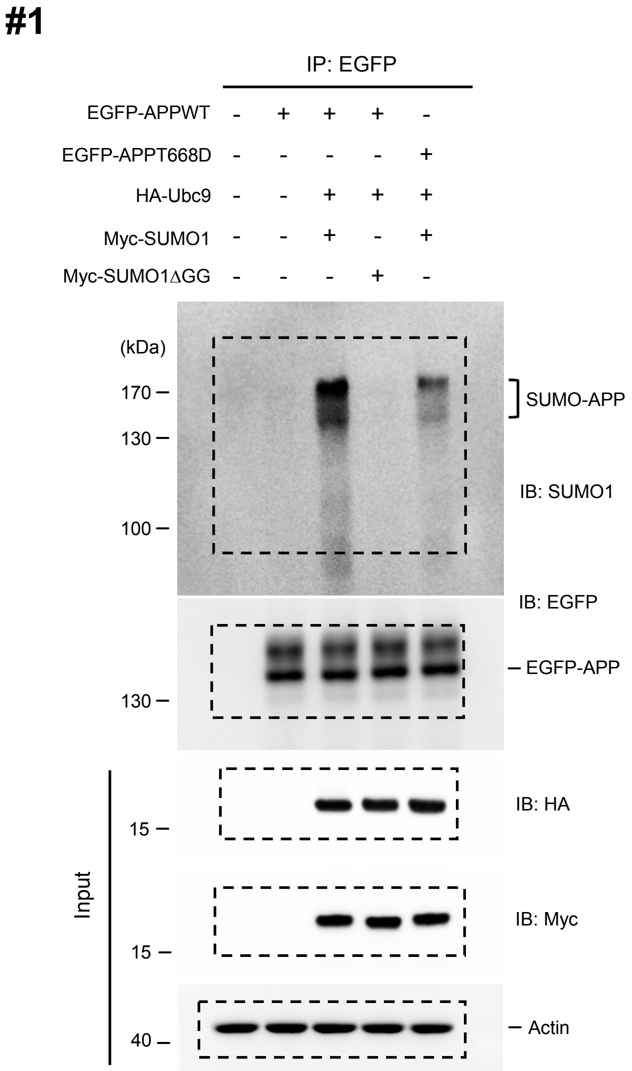

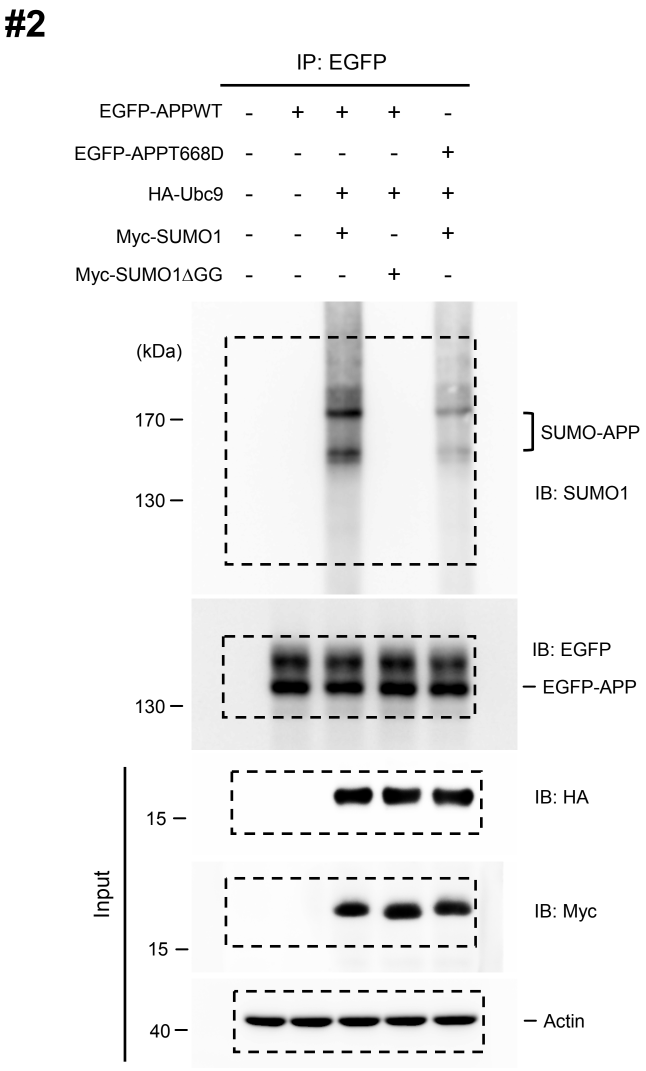

Supplementary Figure 7 original blot

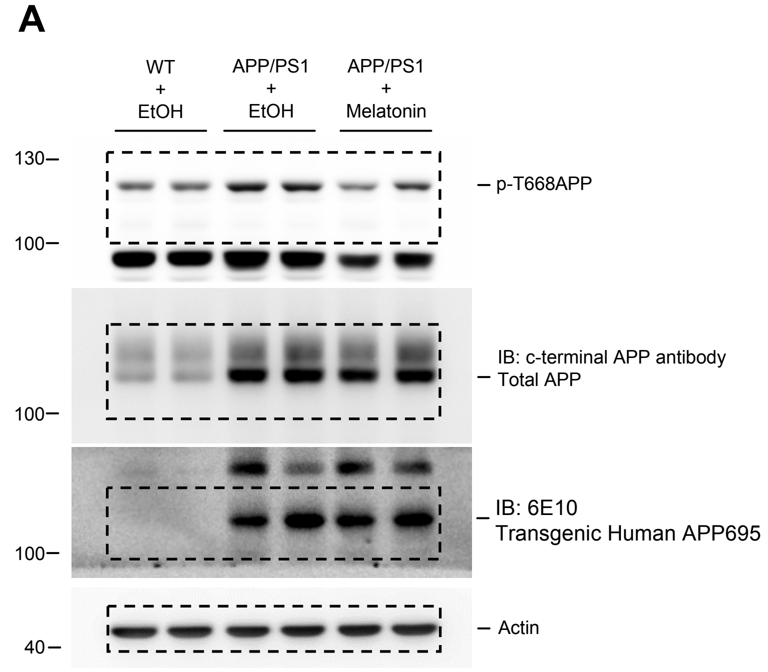

Supplementary Figure 8 original blot

#1

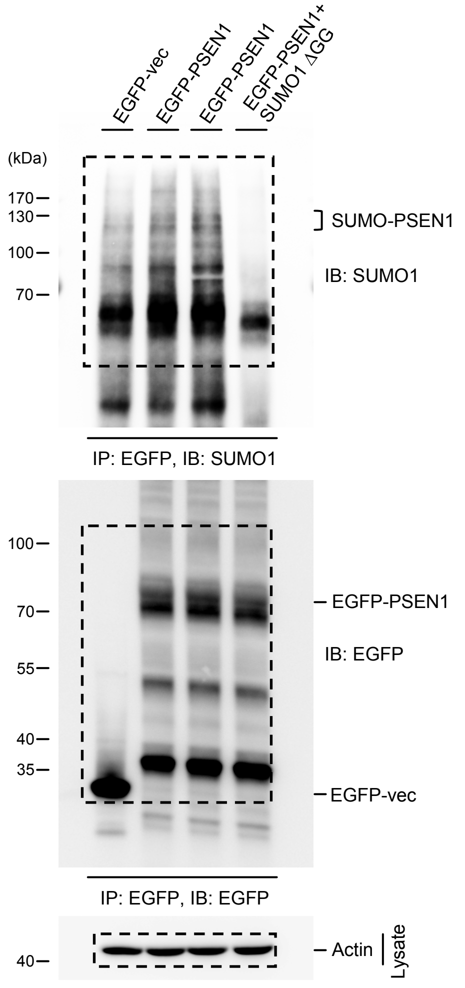

#2

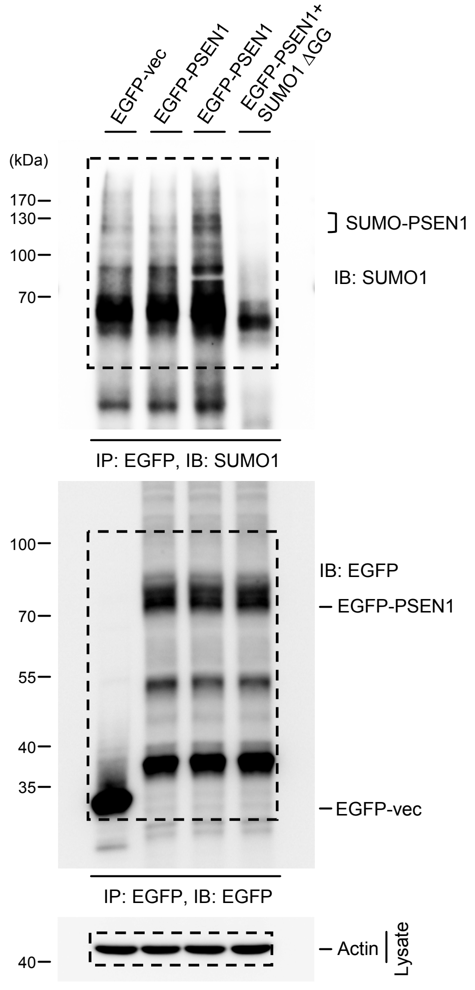

Supplementary Figure 9 (original blot)

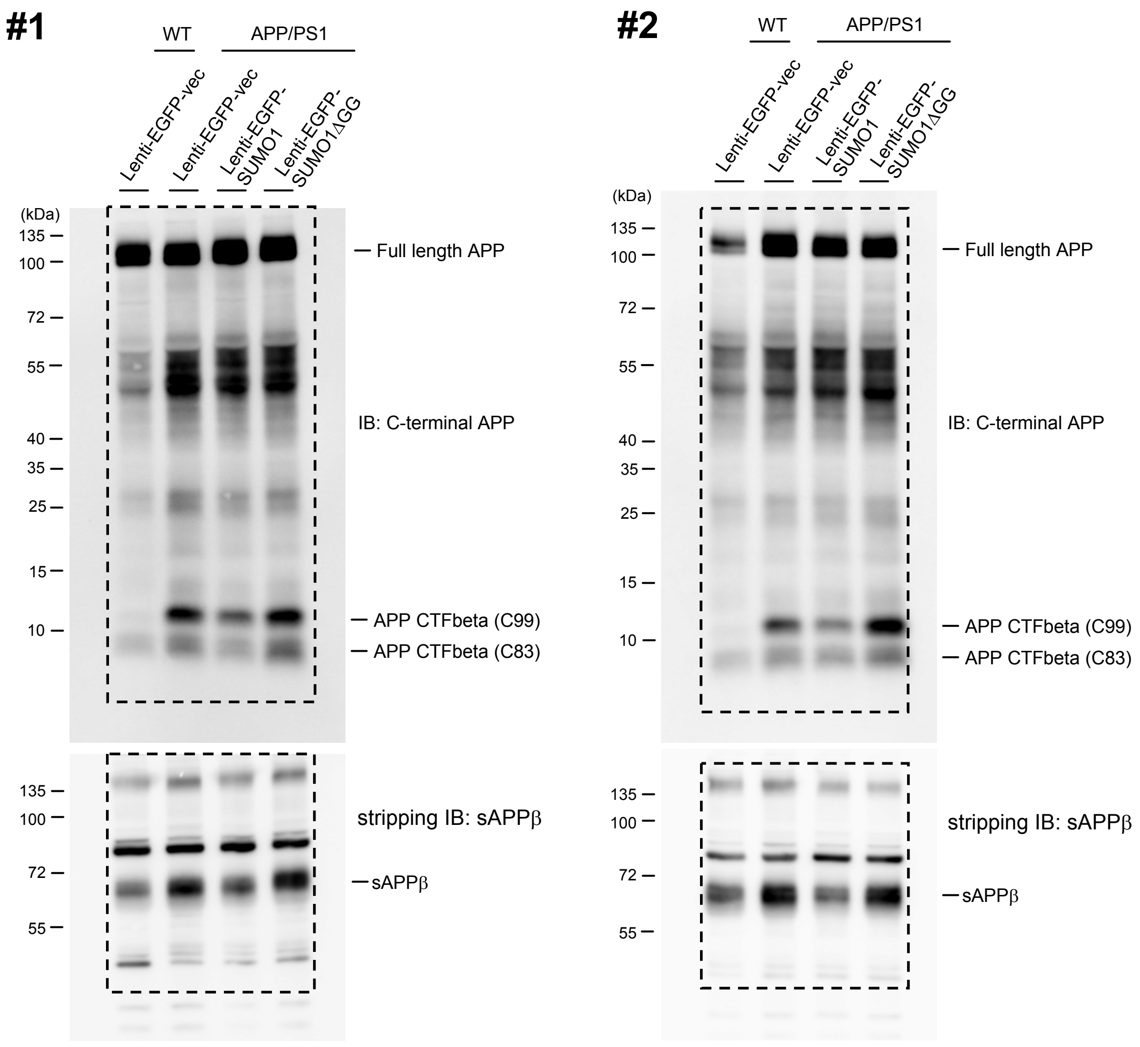

Supplement: Supplementary file 10 — Additional file 10. [file 10020_2025_1354_MOESM10_ESM.pdf]
